# A Serum lncRNA Signature Determines Oncogenic YAP Activity in Cancer Patients

**DOI:** 10.1002/ijc.70584

**Published:** 2026-06-23

**Authors:** Fabian Rose, Nada El‐Ekiaby, Lilija Wehling, Sofia Maria Elisabeth Weiler, Jennifer Schmitt, Marcell Tóth, Fabiola Pedrini, Amruta Damle‐Vartak, Carsten Sticht, Rossella Pellegrino, Injie Omar Fawzy, Merna Hatem Mohamed Hamad, Mohamed Negm, Dina Omar, Hossam Eldeen Soliman, Gamal Esmat, Thomas Longerich, Thomas Illig, Bruno Christian Köhler, Anna Saborowski, Heike Bantel, Arndt Vogel, Peter Schirmacher, Ahmed Ihab Abdelaziz, Kai Breuhahn

**Affiliations:** ^1^ Institute of Pathology Heidelberg University Hospital Heidelberg Germany; ^2^ School of Medicine Newgiza University (NGU) Giza Egypt; ^3^ Core Facility Next Generation Sequencing, Medical Faculty Mannheim Heidelberg University Mannheim Germany; ^4^ Department of Pathology, Kasr Alainy Faculty of Medicine Cairo University Cairo Egypt; ^5^ Department of Hepatobiliary and Pancreatic Surgery, National Liver Institute Menoufiya University Shebin Elkom Egypt; ^6^ Department of Endemic Medicine and Hepatology Cairo University Cairo Egypt; ^7^ Liver Cancer Center Heidelberg Heidelberg University Hospital Heidelberg Germany; ^8^ Hannover Unified Biobank (HUB) Hannover Medical School (MHH) Hannover Germany; ^9^ Department of Medical Oncology, National Center for Tumor Diseases (NCT) Heidelberg University Hospital Heidelberg Heidelberg Germany; ^10^ Department of Gastroenterology, Hepatology and Endocrinology Medical School (MHH) Hannover Germany

**Keywords:** adenocarcinoma, biomarker, hepatocellular carcinoma, liquid biopsy, TAZ

## Abstract

Biomarkers are typically identified by comparing human samples such as tissues and serum. However, reliable markers for transcriptionally active oncogenes remain elusive due to tumor heterogeneity and confounding signals from non‐tumorous cells. We hypothesize that in vitro screening is sufficient to identify a long non‐coding RNA (lncRNA) signature that is a specific marker for oncogenic transcriptional regulators. Exemplified for the Hippo pathway effectors, we integrated experimental NGS and cancer patient expression data with bioinformatics approaches to identify a yes‐associated protein (YAP)‐specific lncRNA signature in hepatocellular carcinoma (HCC) cells. The lncRNAs in this signature include CYTOR, MIR4435‐2HG, SNHG1, and SNHG17, which partly promote HCC cell proliferation and control the sensitivity to YAP/TEA domain transcription factor (TEAD)‐targeted inhibition. In HCC tissues, the lncRNA signature is associated with increased nuclear enrichment of YAP, the expression of YAP target genes, and poor clinical outcomes in patients. This association was also confirmed in cells and tissues of other malignancies, including lung adenocarcinoma (LUAD). Notably, the lncRNA signature is detectable in the serum of HCC patients and predicts YAP activation in tumor tissues.

In summary, the Hippo pathway‐associated lncRNA signature provides a readout for oncogenic YAP activity across cancers, suggesting its potential as a pan‐cancer biomarker. Our results highlight oncogene‐specific lncRNA signatures as valuable tools for diagnostics, therapy selection, and treatment monitoring.

## Abstract (highlights)

What's new?

Long non‐coding RNAs (lncRNAs) signatures are promising cancer biomarkers, since their dysregulation in tumors is reflected in body fluids. In this study, next‐generation sequencing, bioinformatics, and gene expression data were combined to investigate an lncRNA signature linked specifically to the cancer‐promoting YAP in several cancer types, including liver and lung cancer. Analyses show that the lncRNA signature correlates with YAP activation, tumor growth, and poor patient outcomes. Increased signature levels were also linked to sensitivity to YAP‐targeted therapies, suggesting that lncRNA signatures can potentially inform treatment selection and monitoring.

## Graphical abstract

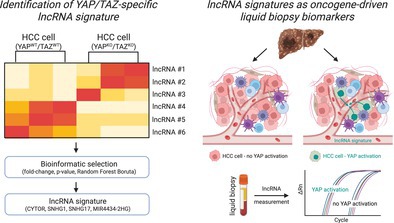

**Abbreviations**
cfDNA: (cell‐free DNA)
cfRNA: (cell‐free RNA)
CTC: (circulating tumor cell)
HCC: (hepatocellular carcinoma)
lncRNA: (long non‐coding RNA)
LUAD: (lung adenocarcinoma)
miRNA: (microRNA)
NGS: (next generation sequencing)
NSCLC: (non‐small cell lung cancer)
TAZ/WWTR1: (WW domain containing transcription regulator 1)
TEAD: (TEA domain transcription factor)
YAP: (yes‐associated protein)

## 1 Background

Robust and sensitive biomarkers are necessary for patient stratification and personalized treatment. In this context, needle biopsy is the gold standard for both conventional and molecular diagnostics. Tissue biomarkers are used for early detection, prognosis assessment, therapy design and monitoring, and identification of risk factors. However, tissue‐based biomarker analysis is often limited by factors such as tissue heterogeneity, dependency on high‐quality antibodies, and the need for invasive sample acquisition. For this reason, biomarker research has begun to focus on liquid biopsy, a minimally invasive and sensitive alternative to tissue biopsy, which enables risk‐free and dynamic monitoring of disease stages [1, 2]. Liquid biopsy analysis enables the identification of gene mutations and typically relies on detecting circulating tumor cells (CTCs) and cell‐free DNA (cfDNA). For example, longitudinal sampling from colorectal cancer (CRC) patients before and after surgery, along with adjuvant chemotherapy, showed that cfDNA could predict tumor relapse [3].

In contrast to cfDNA, which is widely used due to its relatively high stability and established detection methods, cell‐free RNA (cfRNA)—particularly microRNAs (miRNAs) and long non‐coding RNAs (lncRNAs)—has recently gained attention in liquid biopsy approaches. While cfRNA is generally more labile, recent advances in RNA stabilization protocols, blood processing, and sequencing sensitivity have increased its clinical potential, especially for diseases such as liver cancer [4]. Interestingly, studies have shown that circulating lncRNAs, although mostly species‐specific, exhibit sufficient stability and that their dysregulation in tumor tissues is mirrored in corresponding body fluids, making them excellent tools for detecting disease states or even molecular alterations [5, 6].

Due to the lack of robustness and predictive power, only a limited number of liquid biopsy‐based methods have achieved clinical applicability [7]. However, the number of approved liquid biopsy tests is steadily increasing, and their clinical relevance is expected to continue growing in the years to come. Examples include the FDA‐approved diagnostic test for identifying EGFR mutations in lung cancer and monitoring SEPT9 promoter methylation in the plasma cfDNA of colorectal cancer patients [8, 9]. Another example is the identification of PIK3CA mutations in circulating tumor DNA (ctDNA) from patients with metastatic breast cancer and the prediction of response to Alpelisib [10, 11]. To our knowledge, no clinically approved tests exist for measuring lncRNAs in patient samples.

New biomarkers are mainly identified by comparing human tissue or serum through various techniques. Samples from different patient groups are compared, including material collected before and after treatment, at early and late disease stages, and from patients who respond to a specific therapy compared with those who do not. To achieve this, omics technologies, such as next‐generation sequencing (NGS), proteomics, and metabolomics, are employed to characterize these patient groups [12]. However, traditional biomarker identification is restricted by methodological limitations. For instance, tissue‐ or serum‐based omics approaches do not differentiate between the sources of information. In this case, “noise” from non‐tumorous cells may obscure information from diseased cells. Moreover, biomarkers identified in this way do not necessarily offer insights into the activity of non‐mutated yet disease‐relevant genes (e.g., transcription factors activated by signaling pathways). Therefore, alternative approaches are essential for identifying biomarkers specific to oncogene activity in cancer cells.

A signaling pathway that could greatly benefit from specific biomarkers is the Hippo signaling pathway, which includes two oncogenic effectors: yes‐associated protein (YAP) and transcriptional coactivator with PDZ‐binding motif (TAZ). Several lines of experimental evidence demonstrate that the genetic dysregulation of Hippo pathway components or the overexpression of YAP leads to liver tumor formation [13, 14, 15]. Moreover, overexpression and nuclear enrichment of YAP and TAZ are frequently observed in liver, lung, colon, and breast cancer, classifying this pathway as a promising therapeutic target in different tumor types [16, 17]. However, gene mutations in the Hippo pathway that activate these oncogenes are rare, making it difficult to identify robust biomarkers by conventional sequencing [18]. Notably, new inhibitors targeting YAP/TAZ activity have been published or are currently undergoing clinical trials. These inhibitors interfere with the interaction between YAP/TAZ and the TEA domain (TEAD) transcription factors, reducing tumor growth in mouse models (clinical trial IDs: NCT05228015, NCT06251310, NCT04665206) [19, 20, 21]. Consequently, the clinically relevant question arises about which patients might benefit from a YAP/TAZ‐TEAD‐directed therapy.

Illustrated for YAP in hepatocellular carcinoma (HCC) and non‐small cell lung cancer (NSCLC), we highlight the applicability of transcriptionally regulated lncRNA signatures in patient serum as biomarkers of oncogene activity and therapy responsiveness.

## 2 Materials and Methods

Supporting Information describes patient cohorts 1 and 2 (Table S1), cell culture experiments, the mouse model, functional assays, biochemical and molecular analyses, and tissue staining techniques. Antibodies used for western immunoblotting, immunohistochemistry, immunofluorescence staining, and their corresponding dilutions, primers, and siRNA sequences are listed in Tables S2 and S3.

### 2.1 Cell Lines and Culture Conditions

The human HCC cell lines HLF (RRID: CVCL_2947) and Huh‐7 (RRID: CVCL_0336) were obtained from the Japanese Collection of Research Biosources (JCRB, via Tebu‐Bio, Offenbach, Germany). The human lung cancer cell line A‐549 (RRID: CVCL_0023) and the human HCC cell lines SNU‐475 (RRID: CVCL_0497) and SNU‐449 (RRID: CVCL_0454) were obtained from the American Type Culture Collection (ATCC; LGC Standards GmbH, Wesel, Germany). The HCC cell line Hep 3B2.1.7 (RRID: CVCL_0326; also known as Hep3B) was obtained from the German Collection of Microorganisms and Cell Cultures GmbH (DSMZ, Braunschweig, Germany). Cells were cultured in Dulbecco's modified Eagle medium (HLF, Huh‐7, A‐549), Roswell Park Memorial Institute medium 1640 (SNU‐474, SNU‐449), and Minimum Essential Media (Hep 3B2.1.7) supplemented with 10% fetal bovine serum and 1% penicillin/streptomycin (Sigma‐Aldrich, Taufkirchen, Germany). Medium for SNU‐449 cells was additionally supplemented with 1% HEPES, 1% L‐glutamine, and 1% sodium pyruvate. Cells were cultured at 37°C and 5% CO_2_ in a humidified incubator. All experiments were performed with mycoplasma‐free cells and all human cell lines have been authenticated using STR profiling within the last 3 years (Microsynth, Göttingen, Germany). HLF cells were used for the initial screening experiment and verification because YAP/TAZ has a greater transcriptional impact on its target genes in these cells, likely due to a SAV1 truncation mutation (https://lccl.zucmanlab.com/hcc/home) [22, 23].

### 2.2 Patient Samples

This study comprises serum samples from 20 healthy persons and 29 HCC patients (cohort 1 from Hannover Medical School and University Hospital Heidelberg) and 11 healthy individuals and 8 HCC patients (cohort 2 from National Liver Institute, Menoufiya University). Matched paraffin‐embedded HCC samples were available for 17 HCC patients (cohort 1) and 8 HCC patients (cohort 2). Cohort features are listed in Table S1.

Tumor samples used for the HCC tissue microarray (TMA) analysis were surgically resected at the University Hospital of Heidelberg and histologically classified according to established criteria by two experienced pathologists (P.S., T.L.). The TMA contained 40 non‐tumorous liver tissues, 174 cirrhotic liver tissues, and 476 HCCs (grading: G1 = 87, G2 = 311, G3/4 = 78).

Expression data from GDC TCGA cancer cohorts and related clinical features were downloaded from the UCSC Xena database (https://xena.ucsc.edu). K‐means clustering was used to group samples by minimizing within‐cluster variance. Partitioning Around Medoids (PAM) clustering assigned samples to clusters, thereby increasing robustness to outliers.

### 2.3 Expression Profiling

For the identification of YAP and TAZ‐regulated mRNAs and lncRNAs, HLF and A‐549 cells were transfected with two different combinations of YAP and TAZ‐specific siRNAs (40 nM; siYAP/TAZ #1, siYAP/TAZ #2). Total RNA was isolated 24 h after transfection using the NucleoSpin RNA II kit (Macherey‐Nagel, Düren, Germany). Only samples with an RNA integrity number (RIN) > 7 were considered for RNA sequencing (BGI, Hong Kong, China).

RNA‐seq data were analyzed with R and Bioconductor using the package systemPipeR [24]. Quality control of raw sequencing reads was performed using FastQC (https://www.bioinformatics.babraham.ac.uk/projects/fastqc/). Low‐quality reads were removed using trim_galore (version 0.6.4). The resulting reads were aligned to human genome version hg19 from GeneCode and counted using Kallisto version 0.46.1 [25]. The sequencing coverage and quality statistics are summarized in Table S4.

The count data was transformed to log_2_‐counts per million (logCPM), and differential expression analysis was performed using the R package limma. A false positive rate of *α* = 0.05 with FDR correction was taken as the level of significance. Venn diagrams, heatmaps, and volcano plots were created using the R packages VennDiagram [26], ComplexHeatmap [27], and EnhancedVolcano (https://github.com/kevinblighe/EnhancedVolcano), respectively. Raw and normalized data were deposited in the Gene Expression Omnibus database (https://www.ncbi.nlm.nih.gov/geo/; GSE207724).

### 2.4 Expression Data Analysis and Signature Score Calculation

CCLE gene expression data were downloaded from the DepMap portal [28]. Gene expression values were z‐scaled and visualized using the R packages pheatmap (https://CRAN.R‐project.org/package=pheatmap) and ComplexHeatmap.

TCGA gene expression values were *z*‐scaled and discretized into 10 bins using the R packages pheatmap and biclust (https://CRAN.R‐project.org/package=biclust). K‐mean clustering was applied to stratify patients into two groups (lncRNA high and lncRNA low) using the R package ComplexHeatmap. The Random Forest‐based Boruta method was used to calculate the importance of individual lncRNA within the clustering process [29].

Scores for different signatures were calculated for each patient: A lncRNA signature (described here) and two signatures regulated by the Hippo pathway (YAP/TAZ signature #1 and #2) [18, 30]. To ensure that each gene contributes equally to a signature score, single expression values were divided by the mean expression of the corresponding gene in all samples. Equally weighted scores were statistically associated using Spearman's rank correlation coefficient.

### 2.5 Detection of lncRNAs in Serum Samples and Correlation With YAP Activity

Total RNA was isolated from serum samples using the miRNeasy Serum/Plasma Advanced Kit (Qiagen, Hilden, Germany) according to the manufacturer's instructions. In brief, 200 μL serum was transferred into a 2 mL reaction tube. Subsequently, 60 μL RPL buffer was added, vortexed for 5 s, and incubated at RT for 3 min. For sample normalization and to monitor variances in the RNA purification and amplification process, 3 μL of a custom spike‐in control (10 nM) was added to the lysate. About 20 μL RPP buffer was added to samples, vigorously vortexed for 20 s, and incubated at RT for 3 min. Samples were centrifuged at 12,000 g at RT for 3 min to pellet the precipitated nucleic acid. Subsequently, the supernatant was transferred to a new tube and one volume of isopropanol was added. The samples were loaded onto an RNeasy UCP MinElute spin column and centrifuged at 8000 g for 15 s. Samples were washed with 700 μL buffer RWT (8000 g for 15 s), 500 μL buffer RPE (8000 g for 15 s), and 500 μL 80% ethanol (8000 g for 2 min). Columns were dried at full speed for 5 min and the RNA was eluted with 20 μL RNase‐free H_2_O for 2 min.

Reverse transcription was performed with PrimeScript RT Master Mix using 7 μL of total RNA according to the manufacturer's protocol. Subsequently, the lncRNA targets were pre‐amplified using the Prelude PreAmp Master Mix (TakaraBio). For this, equal volumes of each specific primer pair for candidate lncRNAs, negative control lncRNAs, and spike‐in control were added to each sample (final concentration: 500 nM). The pre‐amplification reaction was performed for 14 cycles. Subsequently, a standard qPCR protocol was applied to quantify lncRNA expression levels. Detection of the spike‐in control was used for normalization to account for any technical variances during the isolation process, since housekeeping genes did not allow reproducible sample normalization.

Nuclear YAP expression in HCC tissues was assessed by immunohistochemistry to investigate YAP activity in tissue samples. A score for YAP positivity in nuclei was derived using the following scoring system: 1, not detected; 2, low; 3, moderate; and 4, high. lncRNA signature scores were calculated for serum samples.

### 2.6 Statistical Analysis

Suitable statistical tests were performed using GraphPad Prism 9 software and R version 4.1.0. For each experiment, statistical details can be found in the figure legends, including statistical tests and sample sizes. All in vitro experiments were confirmed by independent biological replicates. Data is represented as mean ± SD. Significance levels are as follows: *p ≤ 0.05, **p ≤ 0.01, ***p ≤ 0.001. Group comparison was performed using the nonparametric Mann–Whitney *U* test. Dunnett's test was employed for multiple tests of functional experiments. For the statistical analysis of the lncRNA signature across different cell lines with varying concentrations of TED‐347, a two‐way ANOVA followed by post hoc Tukey's multiple comparisons was conducted. Patient survival was analyzed using the Kaplan–Meier log‐rank (Mantel–Cox) test or the Gehan–Breslow–Wilcoxon test. The association between data series (e.g., lncRNA signature in serum and YAP positivity in HCC) was calculated using the Spearman correlation coefficient. Receiver operating characteristic (ROC) curve analysis was performed using the R package pROC [31].

## 3 Results

### 3.1 Identification of Hippo Pathway‐Regulated lncRNAs in HCC Cells

**FIGURE 1:**
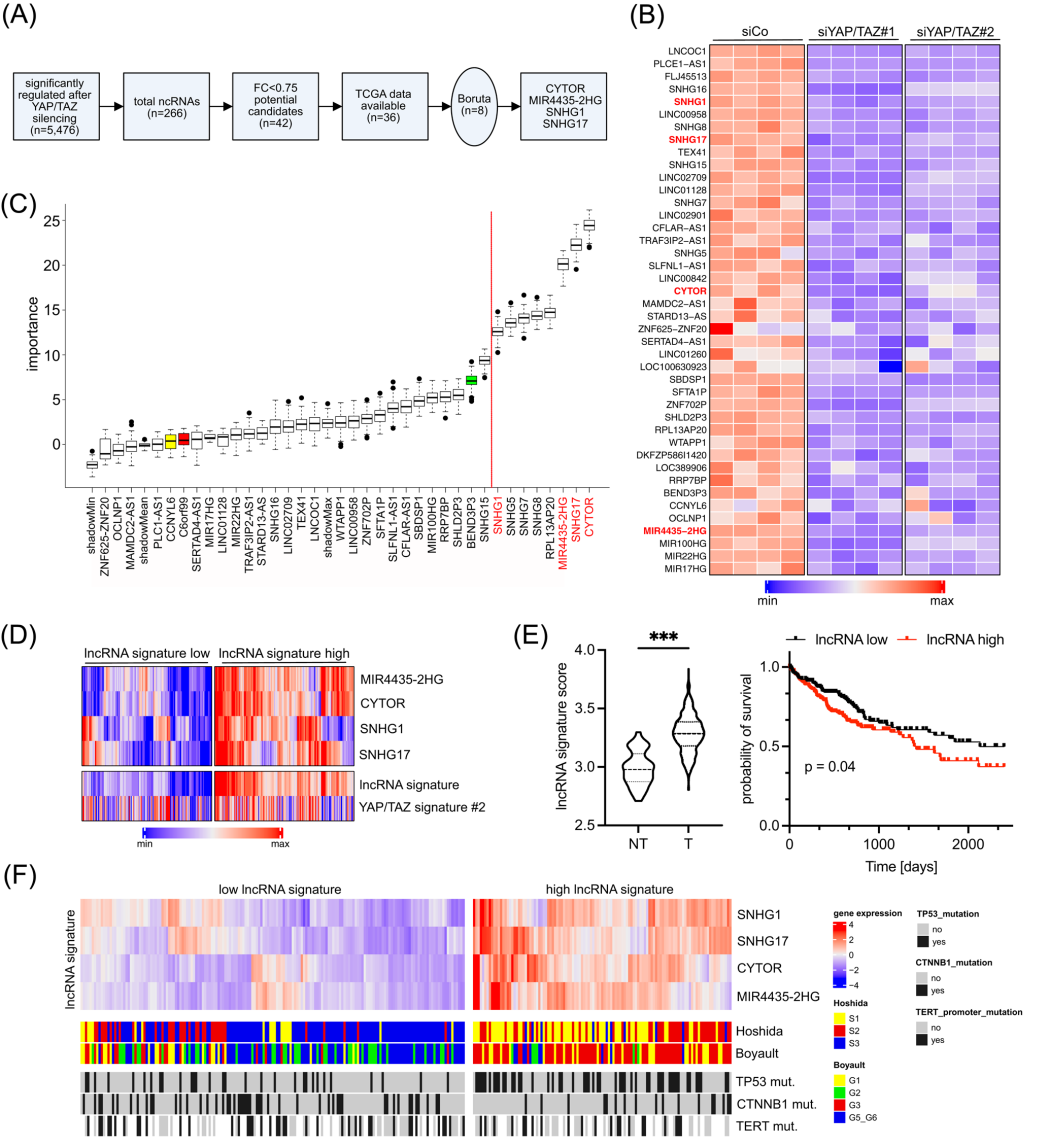
Identification of YAP/TAZ‐regulated lncRNAs in HCC cells. (A) Schematic overview of the processing pipeline that uses experimental NGS data following the combined inhibition of YAP/TAZ in HLF cells. Two siRNA combinations targeting YAP and TAZ were used (siYAP/TAZ #1 and #2). Candidate lncRNAs with available TCGA expression data were used for the Boruta analysis. Four lncRNAs were identified for which primer design was possible (CYTOR, MIR4435‐2HG, SNHG1, and SNHG17). (B) Heatmap summarizing the differential expression of candidate lncRNAs in HCC cells after YAP/TAZ inhibition for 24 h. lncRNAs (*n* = 42) were reduced by at least 0.75 compared with the control reaction. The lncRNA signature constituents are highlighted in red (*n* = 4). (C) The Boruta algorithm for feature selection was applied to identify the most crucial lncRNAs necessary for clustering HCC patients into two groups. Eight lncRNAs exhibited greater importance than the remaining lncRNAs, as indicated by the red line. Features are colored green (confirmed important), red (rejected), or yellow (tentative) based on Boruta's statistical significance against shadow features. Patient data was sourced from the TCGA database. The lncRNA signature constituents are highlighted. (D) Heatmap showing the four signature lncRNAs in HCC patients (*n* = 369, TCGA data). K‐mean clustering was applied to categorize patients into two groups (lncRNA signature low, *n* = 178; lncRNA signature high, *n* = 191). The equally weighted lncRNA signature score and the YAP/TAZ target gene signature #2 are displayed in the lower part of the heatmap. (E) The violin plot illustrates the expression of the lncRNA signature in non‐tumor tissues (NT) and tumor tissues (T). Statistical test: Mann–Whitney *U* test, ***p ≤ 0.001. Kaplan–Meier survival curve for patients with low (lncRNA^low^) and high (lncRNA^high^) signature expression. The *p* value is indicated (Statistical tests: Log‐rank and Mantel–Cox test). (F) PAM clustering was applied to categorize patients into two groups (low lncRNA signature, *n* = 256; high lncRNA signature, *n* = 115). The classification of HCCs according to Hoshida (S1–S3) and Boyault (G1–G6), as well as the mutation status of TP53, CTNNB1, and the TERT promoter are shown.

To illustrate the suitability of genetically modified in vitro systems for identifying biomarkers, we aimed to characterize YAP/TAZ‐dependent lncRNAs in HCC cells. To identify lncRNAs regulated by YAP and TAZ, we analyzed RNA‐seq data from liver cancer cells (Figure 1A). First, we silenced YAP and TAZ using two independent siRNA combinations in HLF cells, as these cells harbor an activating mutation in the upstream regulator Salvador (SAV1) and show pronounced nuclear localization of YAP/TAZ (Figure S1A–C). After inhibition for 24 h, we conducted DNBSEQ NGS on four biological replicates to identify regulated mRNAs and lncRNAs.

Overall, 5476 genes from both siRNA combinations were regulated in the same direction, including 5161 protein‐coding mRNAs. The validity of the siRNA screen in HLF cells was confirmed by the downregulation of two mRNA signatures of known YAP/TAZ target genes (Figure S1D) [18, 30]. Additionally, 266 non‐coding RNAs, composed of pseudo‐ and lncRNAs, were regulated following YAP/TAZ inhibition. Further analysis of lncRNA genes with reduced expression (FC ≤ 0.75) resulted in the identification of 42 YAP/TAZ‐dependent lncRNA candidates (Figure 1B).

To pinpoint the most crucial lncRNAs for classifying patients, we performed a random forest‐based Boruta ranking using expression data from HCC patients (Figure 1C) [32]. This approach allowed the identification of four lncRNAs for which specific primer designs were possible: CYTOR, MIR4435‐2HG, SNHG1, and SNHG17 (Figure 1C, Figure S1E).

Next, we determined the expression of the identified lncRNAs using expression data from HCC patients [32]. For this, we divided patients into groups with high and low expression of all lncRNAs using k‐means clustering (lncRNA signature high and lncRNA signature low, respectively). Indeed, the individual lncRNAs and an equally weighted lncRNA signature score for all four lncRNAs significantly correlated with the presence of YAP target genes (Figure 1D) [18]. In addition, the lncRNA signature showed significant overexpression in HCCs compared to non‐tumorous liver tissues, and its levels correlated with poor patient survival (Figure 1E). The analysis of the individual lncRNAs revealed that, although all of them were significantly overexpressed in the HCC group, only CYTOR showed a statistically significant association with poor survival (Figure S2). This classifies the lncRNA signature as a negative prognostic marker for HCC patients.

Comparison with established molecular classifications of HCC revealed that the lncRNA signature was predominantly expressed in tumors assignable to the S1 subclass (Hoshida) or the G3 subgroup (Boyault) (chi‐square test, *p* < 0.001) (Figure 1F) [33, 34]. The lncRNA signature also correlated with the presence of TP53 mutations (*p* < 0.01) and the absence of CTNNB1 mutations (*p* < 0.05). No significant association was observed with TERT promoter mutations (*p* > 0.05).

In summary, by combining experimental in vitro data with bioinformatic methods, we identified a YAP/TAZ‐regulated lncRNA signature that correlates with poor prognosis in HCC patients.

### 3.2 YAP Transcriptionally Controls the lncRNA Signature Expression in HCC Cells

Further experiments were conducted to confirm the Hippo pathway's specific regulation of the lncRNA signature. As positive controls, we examined the known YAP/TAZ target genes CTGF, ANKRD1, and CYR61, while the YAP/TAZ‐independent lncRNAs identified by our NGS analysis, DLEU1 and FTX, served as negative controls (Figure S3A). First, we verified the results for all four lncRNA candidates using separate siRNA knockdown experiments in independent HCC cell lines by real‐time PCR (HLF, Huh‐7; Figure S3B,C). As indicated by our expression profiling data, the inhibition of YAP/TAZ reduced the CTGF, CYR61, ANKRD1, and all lncRNA signature genes, while the negative control lncRNAs remained unchanged in both cell lines. Inhibition of YAP or TAZ alone generally resulted in no or less pronounced effects, suggesting functional compensation (Figure S3D).

**FIGURE 2:**
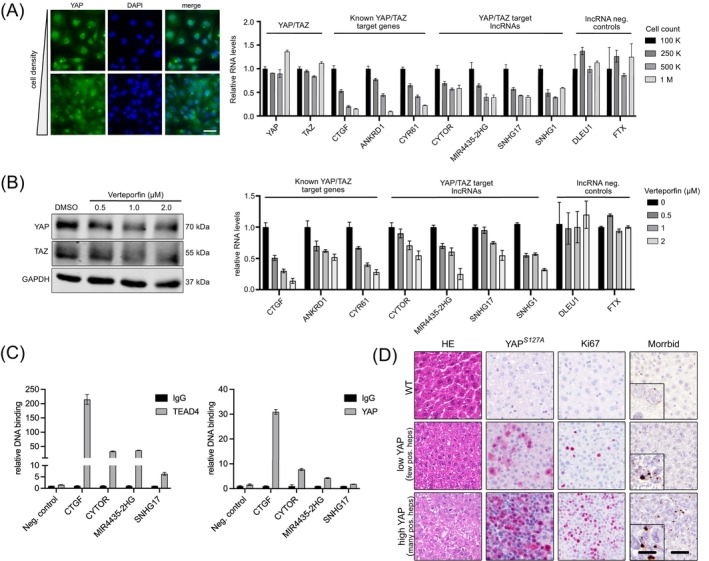
Mechanism of YAP/TAZ‐dependent lncRNA expression in cancer cells. (A) Immunofluorescence images show nuclear YAP exclusion under high cell density growth conditions. qPCR analysis was conducted for YAP/TAZ, its target genes (CTGF, ANKRD1, CYR61), candidate lncRNAs (CYTOR, MIR4435‐2HG, SNHG1, SNHG17), and lncRNAs that are not regulated by YAP/TAZ (DLEU1, FTX). Scale bar: 20 μm. (B) HLF cells were treated with the YAP/TAZ/TEAD inhibitor Verteporfin (0.5–2 μM) for 24 h. Western immunoblots illustrate a concentration‐dependent and expected moderate reduction of both proteins. DMSO served as a control. The genes were analyzed as described before. (C) ChIP experiments were conducted for CTGF (positive control), CYTOR, MIR4435‐2HG, and SNHG17 with TEAD4 and YAP in HLF cells. An upstream promoter region of the CTGF gene was used as a negative control. Robust ChIP primers could not be designed for SNHG1. IgG was utilized as an antibody control. Results were normalized to respective IgG controls. (D) Exemplary hematoxylin/eosin (H&E) stains, immunohistochemistry for YAP^S127A^ and Ki67, and Morrbid in situ hybridization are presented. Specimens were obtained from WT and YAP^S127A^ transgenic mice with low and high numbers of YAP^S127A^‐positive hepatocytes. Samples were collected 6 weeks after DOX administration, resulting in an expansion of YAP‐positive progenitor cells without malignant transformation. Scale bars: Lower magnification: 100 μm; higher magnification: 20 μm.

Next, the cell density‐dependent expression of the candidate lncRNAs was examined, as the Hippo pathway serves as a crucial sensor of cell–cell contact [35]. Indeed, the high‐cell‐density culture conditions led to the exclusion of both transcriptional regulators from the nucleus. This subcellular translocation did not impact the expression of YAP/TAZ but reduced expression of lncRNAs (Figure 2A). As expected, the YAP/TAZ target genes CTGF, ANKRD1, and CYR61 were equally regulated, whereas the lncRNAs DLEU1 and FTX showed no response.

To functionally confirm the TEAD‐dependence of the candidate lncRNAs, we concurrently inhibited TEAD1, TEAD3, and TEAD4 in HCC cells. All signature lncRNAs were regulated by the combined TEAD silencing, while the negative control lncRNAs did not show significant changes (only FTX exhibited a moderate response due to predicted TEAD binding sites in the promoter; Figure S2E). Lastly, treating HLF cells with increasing concentrations of the YAP/TAZ/TEAD inhibitor Verteporfin lowered the expression of the lncRNAs and positive control genes, while the negative controls remained unchanged (Figure 2B) [36].

Subsequent ChIP analysis confirmed that YAP and TEAD4 effectively bind to the predicted TEAD binding sites in the promoters of the SNHG17, CYTOR, and MIR4435‐2HG genes, with CTGF serving as a positive control (Figure 2C, Figure S3F). Notably, TAZ exhibited reduced promoter binding, although it may still contribute to the regulation of canonical YAP/TAZ target genes (Figure S3F). Interestingly, TEAD efficiently binds the promoters of the negative controls DLEU1 and FTX, whereas YAP and TAZ do not interact with these promoters (Figure S3G).

Lastly, we investigated whether the Hippo‐dependent expression of lncRNAs is specific to human cells or whether YAP regulates mouse lncRNA orthologues in liver tissue. To this end, the expression of Morrbid (orthologue for human CYTOR and MIR4435‐2HG), Snhg1, and Snhg17 was studied in a transgenic mouse model that allowed the inducible expression of constitutively active YAP (YAP^S127A^) in hepatocytes [13, 30]. Compared to wildtype (WT) mice, increased levels of Morrbid and Snhg1 were detected in liver lysates 6 or 13 weeks after YAP^S127A^ induction (Figure S3H). Snhg17 did not exhibit a comparable induction. To illustrate cell specificity, in situ hybridization for Morrbid and immunohistochemical staining for YAP were performed, followed by quantitative image analysis. Morrbid levels were elevated in YAP^S127A^ mice and correlated with YAP^S127A^ abundance (*r* = 0.872, *p* = 0.067, *n* = 5) (Figure 2D). Moreover, Morrbid and YAP positivity were associated with the proliferation marker Ki67 (*r* = 0.9487, *p* = 0.067 and *r* = 0.9733, *p* = 0.033, *n* = 5).

These findings demonstrate that YAP and, to a lesser extent, TAZ transcriptionally regulate the expression of lncRNA signature constituents in HCC cells, and that these lncRNAs can serve as surrogate markers.

### 3.3 Tumor Entity‐Spanning Relevance of the YAP/TAZ‐Dependent lncRNA Signature

Using an in vitro screen, we identified a lncRNA signature transcriptionally regulated by YAP. Since lncRNAs are expressed in a highly cell type‐specific manner, we wondered whether a similar relationship exists in other tumor types. For this reason, we conducted a comprehensive investigation of expression data derived from 32 tumor types [32, 37].

**FIGURE 3:**
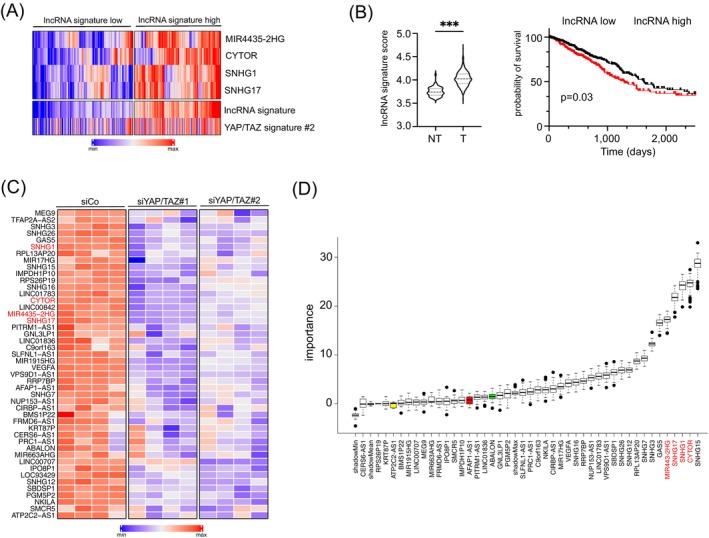
Detection of YAP/TAZ‐regulated lncRNAs in NSCLC/LUAD cells. (A) Heatmap showing the four signature lncRNAs in LUAD patients (*n* = 510). K‐mean clustering was applied to categorize patients into two groups (lncRNA signature low, *n* = 270; lncRNA signature high, *n* = 240). The equally weighted lncRNA signature score and the YAP/TAZ target gene signature #2 are displayed in the lower part of the heatmap. (B) The violin plot illustrates the expression of the lncRNA signature in non‐tumor tissues (NT) and LUAD (T). Statistical test: Mann–Whitney *U* test, ***p ≤ 0.001. Kaplan–Meier survival curve for patients with low (lncRNA low) and high (lncRNA high) signature expression. The *p* value is indicated (Statistical tests: Log‐rank and Gehan‐Breslow‐Wilcoxon). (C) Heatmap summarizing the differential expression of candidate lncRNAs in LUAD cells after YAP/TAZ inhibition for 24 h. The lncRNA signature constituents are highlighted in red. (D) The Boruta algorithm for feature selection was applied to identify the most crucial lncRNAs necessary for clustering LUAD patients into two groups. Some lncRNAs exhibited greater importance than the remaining lncRNAs. The lncRNA signature constituents are highlighted.

K‐means clustering showed that the lncRNA signature in some tumor types effectively discriminated between patient groups and correlated with the YAP/TAZ target gene signature (Table S5). The classification of tumor types into three groups based on the correlation coefficient illustrated that 13 of 32 cancer types exhibited a higher correlation between the lncRNA signature and YAP target genes. Eight of 32 tumor types showed a moderate correlation, while 11 showed a weaker correlation. Importantly, the highest detectable correlation was found in NSCLC, specifically in the lung adenocarcinoma (LUAD) subtype (*r* = 0.5225; *p* ≤ 0.001). As demonstrated in HCC, the YAP‐dependent lncRNA signature is overexpressed in LUAD tissues and correlates with a poorer clinical outcome (Figure 3A,B).

In addition to HCC and LUAD, kidney renal clear cell carcinoma (KIRC) and colon adenocarcinoma (COAD) were examples of a higher statistical association between the lncRNA signature and YAP activity (Table S5; Figure S4A,B). In contrast, other tumor types showed significantly weaker or no association, as exemplified by esophageal carcinoma (ESCA) (Figure S4C).

To experimentally confirm that the lncRNA signature can independently predict YAP/TAZ activity in other cancer types, we repeated the expression profiling analysis after YAP/TAZ inhibition in vitro. For this purpose, we used the LUAD cell line A‐549, as this NSCLC subtype showed the highest correlation in the TCGA expression data (Table S5; Figure S4D). The subsequent selection of lncRNAs along with the application of a random forest‐based Boruta algorithm using LUAD patient data confirmed the presence of CYTOR, MIR4435‐2HG, SNHG1, and SNHG17 among the group of most strongly regulated lncRNAs (Figure 3C,D).

In conclusion, the YAP‐regulated lncRNA signature is present in various tumor types and can act as a robust biomarker to identify cancer patients with YAP/TAZ activity.

### 3.4 Protumorigenic lncRNAs Predict theResponse to YAP/TEAD‐Directed Perturbation

Dysregulation of YAP has been described as a key driver of liver cancer [38], enhancing cell proliferation in HCC cells [39]. Similarly, many lncRNAs promote pro‐tumorigenic functions across various tumor types [40]. Thus, we asked if the YAP‐regulated lncRNAs could support tumor growth and indicate whether tumor cells respond to YAP‐targeted inhibition.

**FIGURE 4:**
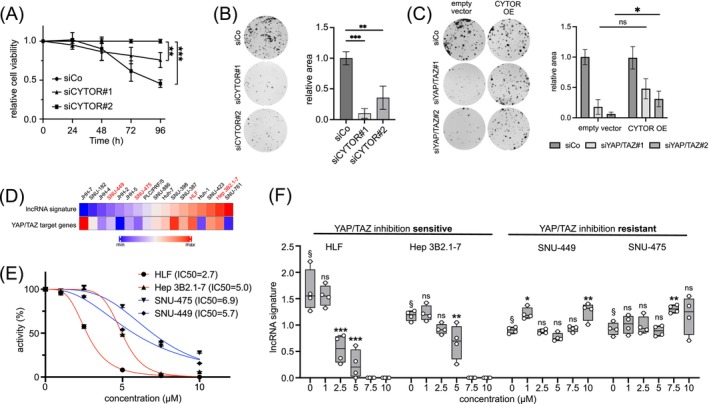
Signature lncRNAs support tumor growth and predict responsiveness to YAP/TAZ/TEAD‐directed perturbation. (A) Cell viability assay using Resazurin in HLF cells after CYTOR inhibition with two siRNAs at the specified time points. (B) A colony formation assay was conducted using HLF cells after 10 days of siRNA‐mediated silencing of CYTOR. (C) Colony formation assay was performed using HLF cells with stable overexpression of CYTOR following siRNA‐mediated knockdown of YAP/TAZ. HLF cells stably transfected with an empty vector served as a control. OE: Overexpression. (D) Heatmap indicating the presence of the equally weighted lncRNA signature score (consisting of *CYTOR, SNHG1, SNHG17, MIR4435‐2HG*) and a YAP/TAZ target gene score in 17 HCC cell lines (CCLE expression data) [18]. Cell lines with high (HLF, Hep 3B2.1.7) and low (SNU‐449, SNU‐475) lncRNA signature and target gene scores were used for subsequent analyses. (E) The graph illustrates a cell viability assay for HLF, Hep 3B2.1.7, SNU‐449, and SNU‐475 cells after 48 h of treatment with different concentrations of the YAP/TEAD inhibitor TED‐347. IC50 values are indicated. (F) Expression of lncRNA signature in sensitive and resistant HCC cell lines after treatment with TED‐347. Drug concentrations are indicated. For sensitive cells, measurements at 7.5 and 10 μM of TED‐347 were not feasible due to adverse effects on cell viability. Graphs A, B, and C summarize the results of three independent experiments/biological replicates. Cells transfected with scrambled siRNA (siCo) served as controls and were used for normalization. For each experiment, two independent siRNA combinations were used (#1, #2). Statistical test (A, B, and C): Dunnett's multiple comparison test. Statistical test (F): Two‐way ANOVA followed by Turkey's multiple comparisons test. *p ≤ 0.05, **p ≤ 0.01, ***p ≤ 0.001.

We first tested if the identified lncRNAs contribute to the tumor‐supporting phenotype driven by YAP. siRNA‐mediated inhibition of MIR4435‐2HG, CYTOR, SNHG17, and SNHG1 by siRNAs significantly reduced cell viability, colony formation, and proliferation in different HCC cell lines (Figure 4A,B; Figure S5A–E). Only for SNHG1 was a discrepancy observed between HLF and Huh‐7 cells. While SNHG1 knockdown reduced all investigated parameters in HLF cells, no obvious effects were detectable in Huh‐7 cells (Figure S5D).

Exemplified for CYTOR, a rescue experiment revealed that its overexpression significantly restored colony formation following combined YAP/TAZ silencing (Figure 4C). These effects on colony formation and CYTOR‐mediated rescue were absent or only marginal following single inhibition of either YAP or TAZ (Figure S5F). Thus, the identified lncRNAs contribute to most of the protumorigenic properties of combined YAP/TAZ dysregulation in HCC cells.

Next, we explored in vitro whether the lncRNA signature indicates the responsiveness of HCC cells to YAP/TAZ/TEAD‐targeted inhibition. To achieve this, we selected cell lines with high (HLF, Hep 3B2.1.7) and low (SNU‐449, SNU‐475) lncRNA signature scores and YAP activity as indicated by YAP/TAZ target gene expression (Figure 4D). Then, we evaluated the sensitivity of these cell lines to the YAP/TEAD inhibitor TED‐347 [41]. Cells exhibiting higher lncRNA signature or YAP/TAZ target gene signature expression demonstrated greater sensitivity to TED‐347 than those with lower lncRNA signature levels, as shown by the IC50 values (Figure 4E). Subsequent measurements of the lncRNA signature also revealed significantly different treatment responses between sensitive and resistant cell lines (Figure 4F). Post hoc analysis showed a significant decrease in the lncRNA signature at lower concentrations in sensitive cells following TED‐347 treatment. In contrast, cell lines exhibiting low expression of lncRNA signatures did not show significantly decreased expression but were characterized by no expression or even elevated signature expression.

These results illustrate that the identified lncRNAs in part facilitate the tumor‐supporting properties of YAP. Additionally, the data underscore the potential of the lncRNA signature in monitoring treatment responses to YAP/TAZ‐directed therapies in HCC cells.

### 3.5 Expression of YAP/TAZ‐Dependent lncRNAs in HCC Tissue

**FIGURE 5:**
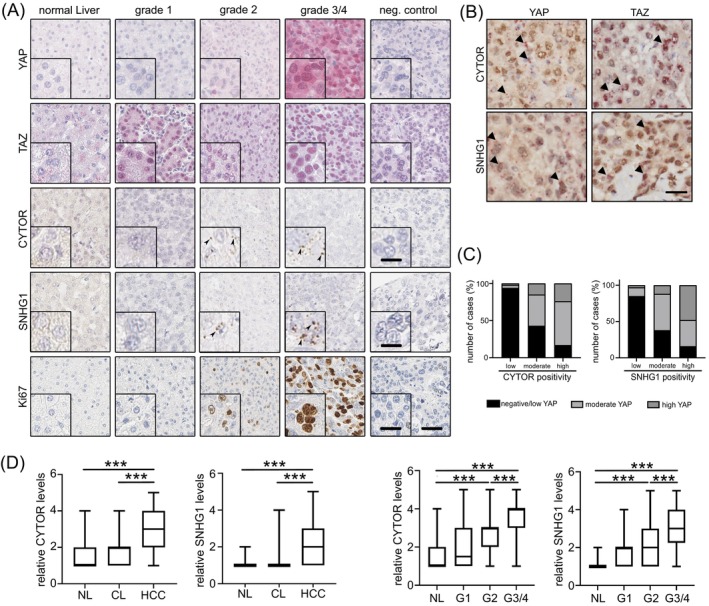
YAP‐dependent lncRNAs are expressed by tumor cells in HCC tissues. (A) IHC stains (YAP, TAZ, Ki67) and in situ hybridization (CYTOR and SNHG1) using human HCC tissue microarrays (40 nontumorous liver tissues, 174 cirrhotic liver tissues, and 476 HCCs, including G1 = 87, G2 = 311, G3/4 = 78). Representative liver tissues and HCCs for well (G1, G2) and poorly differentiated (G3/G4) tumors and a YAP‐negative specimen are shown. Scale bar: Lower magnification: 100 μm; intermediate magnification: 20 μm; highest magnification (in situ inserts): 5 μm. (B) Combined detection of YAP and TAZ by IHC and of CYTOR and SNHG1 by in situ hybridization in HCC tissues. Arrows point to clusters of lncRNAs in tumor cells. Scale bar: 25 μm. (C) Contingency analysis and graphical representation of the associations between nuclear YAP positivity and the expression of the lncRNAs CYTOR and SNHG1. (D) The left boxplots show the distribution of in situ hybridization signals in normal livers (NL), cirrhotic livers (CL), and HCC samples for CYTOR (left) and SNHG1 (right). The right boxplots depict the distribution of in situ hybridization signals in NL and HCC samples with increasing tumor dedifferentiation (NL, G1, G2, and G3/4).

To demonstrate that tumor cells are the source of YAP/TAZ‐dependent lncRNAs in human hepatocarcinogenesis, we conducted in situ hybridization for lncRNAs using probes for CYTOR and SNHG1 on TMAs, which included 40 non‐tumorous liver tissues, 174 cirrhotic liver tissues, and 476 HCCs. Positivity for CYTOR and SNHG1 was mainly detected in tumor cells rather than in hepatocytes or non‐parenchymal cells of the liver, such as Kupffer cells or liver sinusoidal endothelial cells (Figure 5A,B). The expression levels of both lncRNAs were significantly higher in HCCs compared to histologically normal livers or cirrhotic livers, and they correlated with tumor dedifferentiation (Figure 5D). Subsequent immunohistochemical YAP and TAZ stainings revealed a strong correlation of CYTOR and SNHG1 with nuclear YAP in HCC cells (*r* = 0.71, *p* ≤ 0.001; *r* = 0.65, *p* ≤ 0.001). In contrast, only a moderate association was observed between both lncRNAs and nuclear TAZ (*r* = 0.38, *p* ≤ 0.001; *r* = 0.45, *p* ≤ 0.001). Focusing on YAP, a contingency analysis comparing nuclear YAP positivity with the expression levels of the lncRNAs CYTOR and SNHG1 (categorized into tertiles) revealed a significant association (chi‐square test, *p* < 0.001) (Figure 5C). In addition, CYTOR and SNHG1 statistically correlated with the proliferation marker Ki67 (*r* = 0.49, *p* ≤ 0.001; *r* = 0.61, *p* ≤ 0.001, respectively).

These data demonstrate that liver tumor cells express higher levels of lncRNAs and represent the primary source of YAP/TAZ‐induced lncRNAs.

### 3.6 The lncRNA Signature in Serum Serves as a Biomarker for YAP Activity in Cancer Cells

We hypothesize that detecting oncogene‐regulated lncRNAs in patients' serum can serve as promising markers for oncogene activity in tumor tissues. To evaluate the biomarker potential of our lncRNA signature, we measured all four lncRNAs in serum samples obtained from healthy individuals (*n* = 20) and HCC patients (*n* = 29, cohort 1) using semiquantitative real‐time PCR.

For all individual signature lncRNAs, a significantly elevated expression was detectable in sera of HCC patients (Figure S6A). As expected, the increase of lncRNAs was not seen in all HCC cases because YAP activation occurs in only a subset of HCC patients [30]. Accordingly, the equally weighted lncRNA signature score was significantly higher in the sera of HCC patients compared to healthy donors (Figure S6B). For the HCC patients with available formalin‐fixed and paraffin‐embedded material containing viable tumor cell content, YAP immunohistochemistry staining was performed (*n* = 17) (Figure S6C). Notably, correlating the relative abundance of the lncRNA signature in serum with YAP positivity in tumor cells revealed a significant positive association (*r* = 0.75, *p* ≤ 0.001) (Figure S6D). The negative control lncRNAs DLEU1 and FTX consistently failed to correlate with nuclear YAP (*r* = 0.006, *p* = 0.982, Figure S6E).

**FIGURE 6:**
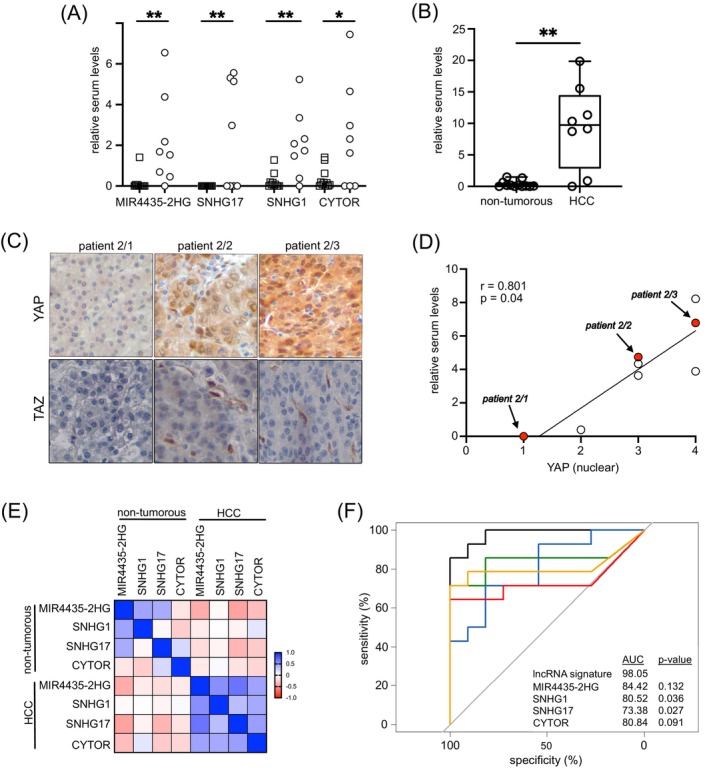
Signature lncRNAs in serum correlate with YAP expression in HCC cells. (A) qPCR analysis of signature lncRNAs in the serum of healthy individuals (*n* = 11, rectangles) and HCC patients (*n* = 8, circles) (cohort 2). Statistical test: Mann–Whitney *U*. ***p ≤ 0.001. (B) Boxplot illustrating the balanced lncRNA signature score in serum derived from healthy individuals and HCC patients (cohort 2). Statistical test: Mann–Whitney *U*. ***p ≤ 0.001. (C) Representative YAP and TAZ stains of cohort 2 HCC patients exhibiting low (Patient 2/1), intermediate (Patient 2/2), and high nuclear YAP abundance (Patient 2/3). (D) The graph illustrates the relationship between lncRNA signature levels in serum and the abundance of nuclear YAP (values ranging from 1 to 4) in the corresponding tissue specimens. Line: Linear regression. Patients 2/1, 2/2, and 2/3 are highlighted. Statistical test: Spearman correlation. The *p* value is indicated. (E) Correlation map of lncRNAs measured in serum from healthy individuals and HCC patients (combined cohorts 1 and 2). A stronger association among signature lncRNAs is detectable in the serum of cancer patients but not in healthy individuals. (F) ROC curve analysis comparing the performance of the serum lncRNA signature and individual lncRNAs in predicting YAP activity/nuclear enrichment within the tissues of HCC patients (combined cohorts 1 and 2, *n* = 25). Statistical test: Two‐sided DeLong's test. The *p*‐values are indicated.

Due to the time gap between serum and tissue sampling in some patients of cohort 1 (Table S1), we conducted a new prospective study involving HCC patients, in which serum and tissue samples were collected concurrently (11 healthy individuals and 8 HCC patients, cohort 2). As described for cohort 1, all lncRNAs in cohort 2 and their respective signature were elevated in the sera of many cancer patients (Figure 6A,B). The statistical correlation between lncRNA signature expression in serum and nuclear YAP in tumor cells was even stronger in cohort 2, despite the smaller number of analyzed patients (*r* = 0.8, *p* = 0.004, Figure 6C,D). In contrast, the statistical analysis between TAZ positivity in the tumors and the lncRNA signature in the serum revealed no significant correlation (*r* = 0.15, *p* = 0.7) (Figure 6C).

Combining the cancer patient data of cohorts 1 and 2, the Spearman correlation analysis revealed a significant statistical association among all four lncRNAs in HCC but not in non‐tumorous liver tissues (Figure 6E). This indicated that many HCC patients exhibited coordinated elevations in all measured lncRNAs, whereas no such pattern was observed in healthy individuals.

Finally, we established the predictive power of the lncRNA signature and combined HCC cohorts 1 and 2 to conduct a ROC analysis. In comparison to the negative controls DLEU and FTX, the individual lncRNA signature demonstrated a superior predictive ability for the presence of nuclear YAP in HCC tissue samples (e.g., AUC_lncRNA signature_ = 98.05 vs. AUC_SNHG17_ = 73.38) (Figure 6F).

These results demonstrate that serum lncRNA signatures can function as biomarkers, predicting the presence and activity of the oncogene YAP in HCC cells.

## 4 Discussion

Current biomarker identification in oncology usually depends on comparing patient material from predefined groups. These groups can include diseased versus healthy individuals, responders versus non‐responders, patients with early‐stage versus late‐stage disease, and those with or without recurrence. Thus, comparing patient material can yield excellent biomarkers that differentiate patient groups. However, determining disease‐relevant mechanisms or identifying potential therapeutic targets is not immediately feasible. Such disease‐relevant mechanisms can include, for example, the mutation‐independent activation of signaling pathways and their transcriptional regulators. For many of these cellular pathways and their constituents, inhibitors exist, or initial compounds are currently being tested in clinical trials.

Our study expands on previous concepts of biomarker identification, as human material is not used for the initial screening but rather for confirmatory purposes. The use of cell lines or, if available, organoids for an initial biomarker screening offers several advantages: (1) In vitro systems are genetically manipulable, directly associating differentially expressed biomarkers with inhibited or overexpressed factors. (2) Biomarkers that might not have been shortlisted in tissue comparisons due to moderate expression levels can play a significant role as pathway‐specific markers. (3) Using cell lines derived from diseased tissue guarantees the accurate assignment of the biomarker to the corresponding cell type. Non‐tumor cells do not provide irrelevant information during biomarker identification. (4) In vitro models are easy to handle and have no limitations regarding availability. We have utilized human tissues only for hypothesis validation, ensuring resource efficiency. Notably, an important limitation of in vitro‐based analyses is that cell lines are usually cultured for many years. This prolonged cultivation typically leads to adaptations and alterations of the cellular system. These include, for example, metabolic adaptations, epigenetic changes, loss of cell‐matrix interactions, and genetic instability [42, 43, 44]. Using organoids derived from primary tissues may help resolve these issues [45].

Despite potential limitations, we identified a robust lncRNA signature in HCC cell lines, whose expression is predominantly controlled by the transcriptional regulator YAP. Although TAZ appears to play a central role in vitro, where functional compensation upon inhibition of one factor is observed, its impact is less pronounced in the tissue context. This relationship was demonstrated through correlative analyses (e.g., in the tissue context) and mechanistic investigations (e.g., ChIP). Furthermore, we independently confirmed the association between YAP activity and lncRNA signature expression in NSCLC/LUAD (experimentally and at the tissue level) and other tumor types (at the tissue level). Thus, the lncRNA signature we identified could serve as a robust biomarker for YAP activity across different tumor types despite lncRNAs being described as highly cell‐type‐specific [46]. It is tempting to speculate that most lncRNAs are subject to cell‐ and development‐specific regulation, making them unsuitable as cancer‐spanning biomarkers. However, some sets of lncRNAs may be so robustly regulated by specific transcription factor complexes that this regulation can be observed across various cell types. In this context, these groups of lncRNAs could act as a promising tool for future studies on transcription factor‐specific biomarkers, extending the findings of this study.

Interestingly, the association between lncRNA signature expression and YAP target gene expression is not detectable in all tumor types. This is the case, for example, in ESCA and cholangiocarcinoma (CHOL/CCC) of the liver. The lack of correlation in CCC tissues is particularly surprising, as YAP is known to promote the dedifferentiation of hepatocytes and the development of tumors with cholangiocellular features [47, 48]. The weak association between the lncRNA and mRNA signatures—both of which would be induced by YAP—may have different explanations. First, the use of bulk tissue for expression analysis may lead to confounding effects from non‐tumorous cell populations such as infiltrating immune cells. Second, cell type‐specific—and so far not identified—transcription factors may influence the expression of lncRNAs or YAP target genes [49].

Different publications demonstrate the regulatory role of individual lncRNAs in cellular signaling and the activity of transcriptional regulators, as illustrated for c‐MYC or β‐catenin [50, 51]. However, these studies discuss lncRNAs as cellular modulators while overlooking their significance as biomarkers for abnormal transcription factor activity. Vice versa, for many transcription factor complexes such as c‐MYC and YAP/TEAD, individual lncRNAs have been identified as target genes, downstream effectors, and/or biomarkers in specific cancer types [52, 53]. Indeed, even sets of lncRNAs have been described as potential biomarkers. For example, a panel of three serum lncRNAs was discussed as diagnostic biomarkers for HBV‐related HCC [54]. Furthermore, a nine‐lncRNA gene signature was described as a biomarker for distant metastasis of nasopharyngeal carcinoma [55]. However, the potential of experimentally identified lncRNA signatures to reflect the activity of oncogenes in various tumor types and their detection in patients' serum, along with the high sensitivity and specificity of these signatures, has not yet been demonstrated.

This approach has a high translational potential. Even in tumors with high genetic heterogeneity, different transcription factor‐specific lncRNA signatures could assist oncologists in creating personalized therapies (e.g., by combining inhibitors of pathways that control the respective transcriptional modules). Additionally, frequent longitudinal serum sampling permits the monitoring of therapeutic success, as indicated by the decrease of the lncRNA signature after treatment of sensitive HCC cells with TED‐347 in our study [41]. It would even allow the detection and genetic characterization of resistant tumor cells before imaging techniques can confirm tumor relapse. In general, the early detection of alternative lncRNA signatures would allow an immediate adjustment of therapy strategies to detect tumor relapse or prevent cancer progression.

## Author Contributions

**Fabian Rose:** formal analysis, investigation, visualization, writing – original draft, writing – review and editing. **Nada El‐Ekiaby:** investigation, formal analysis, writing – review and editing, visualization. **Lilija Wehling:** software, investigation, visualization, writing – review and editing, methodology. **Sofia Maria Elisabeth Weiler:** methodology, writing – review and editing, supervision. **Jennifer Schmitt:** writing – review and editing, formal analysis. **Marcell Tóth:** investigation, writing – review and editing, methodology. **Fabiola Pedrini:** investigation, validation, writing – review and editing. **Amruta Damle‐Vartak:** supervision, writing – review and editing, investigation. **Carsten Sticht:** methodology, investigation, writing – review and editing. **Rossella Pellegrino:** investigation, writing – review and editing, resources. **Injie Omar Fawzy:** investigation, writing – review and editing, methodology. **Merna Hatem Mohamed Hamad:** writing – review and editing, investigation, methodology. **Mohamed Negm:** writing – review and editing, investigation, validation. **Dina Omar:** methodology, writing – review and editing, investigation. **Hossam Eldeen Soliman:** resources, writing – review and editing. **Gamal Esmat:** writing – review and editing, resources. **Thomas Longerich:** writing – review and editing, methodology, resources. **Thomas Illig:** resources, writing – review and editing. **Bruno Christian Köhler:** resources, writing – review and editing. **Anna Saborowski:** resources, writing – review and editing. **Heike Bantel:** resources, writing – review and editing. **Arndt Vogel:** resources, writing – review and editing. **Peter Schirmacher:** writing – review and editing, project administration, resources. **Ahmed Ihab Abdelaziz:** resources, project administration, supervision, writing – review and editing, writing – original draft, funding acquisition. **Kai Breuhahn:** writing – review and editing, writing – original draft, funding acquisition, visualization, project administration, supervision, formal analysis, conceptualization.

## Funding

K.B., A.I.A., and B.C.K. received funding from the Deutsche Forschungsgemeinschaft (D.F.G.; 412294938 to K.B./A.I.A., 505755359 to K.B., 469903177 to B.C.K.). K.B. was financially supported by the German Cancer Aid (70114966). N.E.E. and A.I.A. received financial support from the Alexander von Humboldt‐Stiftung (EGY‐1160930, EGY‐1207096). A.I.A. received financial support from the Science and Technology Development Fund (STDF) under grant number BARG 37096.

## Disclosure

Parts of this manuscript are based on the doctoral dissertation of Dr. Fabian Rose, titled “A pan‐cancer long non‐coding RNA (lncRNA) signature defines oncogene activity in blood serum of cancer patients” submitted to Ruprecht‐Karls‐University Heidelberg in 2023 [56].

## Ethics Statement

All animal experiments were performed under pathogen‐free conditions, following the institutional regulations of the IBF (Interfakultäre Biomedizinische Forschungseinrichtung, University of Heidelberg; approval by the Regional Council of Karlsruhe, ref. number: G‐65/14). The respective institutional ethics committees of Hannover (3434‐2016, 1818‐2013), Heidelberg (S‐206/2005, S‐428/2013, S‐359/2018), and Cairo (00163/2019) approved the use of patient material (tissue and serum) for research purposes. The institutional ethics committee of the Medical Faculty of Heidelberg University also approved the use of samples for the HCC tissue microarray (S‐206/2005). Informed consent has been obtained from all patients.

## Conflicts of Interest

The authors declare no conflicts of interest.

## Supporting information


**Table S1:** Characteristics of cohort 1 and 2.
**Table S2:** Oligos used in the manuscript.
**Table S3:** antibodies used in the manuscript.
**Table S4:** RNA sequencing of HLF cells (GSE207724 subseries GSE207722).
**Table S5:** Correlation of four lncRNA signature with the YAP‐dependent mRNA signature #2 in 32 tumor types (TCGA database for which lncRNA data is available).
**Figure S1:** Identification of YAP/TAZ‐regulated lncRNAs in HCC cells.
**Figure S2:** Expression of lncRNAs in HCC patients.
**Figure S3:** Mechanism of YAP/TAZ‐dependent lncRNA expression in cancer cells.
**Figure S4:** Detection of YAP/TAZ‐regulated lncRNAs in other cancer types.
**Figure S5:** Pro‐tumorigenic function of YAP/TAZ‐induced lncRNAs.
**Figure S6:** Signature lncRNAs in serum correlate with YAP expression in HCC cells.

## Data Availability

Raw and normalized NGS data for HLF and A‐549 cells were deposited in the Gene Expression Omnibus database (https://www.ncbi.nlm.nih.gov/geo/; GSE207724 with the subseries GSE207722 and GSE207723). Expression data from GDC TCGA cancer cohorts and related clinical features were downloaded from the UCSC Xena database (https://xena.ucsc.edu). The data used in this study are derived from the TCGA Research Network (https://www.cancer.gov/about‐nci/organization/ccg/research/structural‐genomics/tcga). Further information is available from the corresponding author upon request.
