# Supplementary material for: A Serum lncRNA Signature Determines Oncogenic YAP Activity in Cancer Patients
Source: Int J Cancer. 2026 Jun 23;159(7):1829–43. doi: 10.1002/ijc.70584 (PMC13432287; doi:10.1002/ijc.70584)
Supplement: Supplementary file 1 — Table S1: Characteristics of cohort 1 and 2. Table S2: Oligos used in the manuscript. Table S3: antibodies used in the manuscript. Table S4: RNA sequencing of HLF cells (GSE207724 subseries GSE207722). Table S5: Correlation of four lncRNA signature with the YAP‐dependent mRNA signature #2 in 32 tumor types (TCGA database for which lncRNA data is available). Figure S1: Identification of YAP/TAZ‐regulated lncRNAs in HCC cells. Figure S2: Expression of lncRNAs in HCC patients. Figure S3: Mechanism of YAP/TAZ‐dependent lncRNA expression in cancer cells. Figure S4: Detection of YAP/TAZ‐regulated lncRNAs in other cancer types. Figure S5: Pro‐tumorigenic function of YAP/TAZ‐induced lncRNAs. Figure S6: Signature lncRNAs in serum correlate with YAP expression in HCC cells. [file IJC-159-1829-s001.pdf]

A serum lncRNA signature determines oncogenic YAP activity in cancer patients

Fabian Rose, Nada El-Ekiaby, Lilija Wehling, Sofia Maria Elisabeth Weiler, Jennifer Schmitt, Marcell Tóth, Fabiola Pedrini, Amruta Damle-Vartak, Carsten Sticht, Rossella Pellegrino, Injie Omar Fawzy, Merna Hatem Mohamed Hamad, Mohamed Negm, Dina Omar, Hossam Eldeen Soliman, Gamal Esmat, Thomas Longerich Thomas Illig, Bruno Christian Köhler, Anna Saborowski, Heike Bantel, Arndt Vogel, Peter Schirmacher, Ahmed Ihab Abdelaziz, Kai Breuhahn

Table of content

1) Supplementary Materials and Methods

2) Supplementary Tables S1-S5

3) Supplementary Figures S1-S6

4) Supplementary References

Supplementary Materials & Methods

A serum lncRNA signature determines oncogenic YAP activity in hepatocellular carcinoma patients

Mouse model

All experiments were performed in accordance with the institutional regulations of the IBF (Interfakultäre Biomedizinische Forschungseinrichtung, University of Heidelberg) under pathogen-free conditions. The mouse colony was housed under a 12-hour light/dark cycle with free access to water and food. Exclusion and termination criteria were defined in the ATBW criteria (officials for animal welfare).

The LAP-tTA/Col1A1-YAP^{S127A} transgenic mouse models for the inducible expression of constitutively active human YAP^{S127A} were used for this study.[1, 2] For transgene induction, doxycycline (DOX, 2 mg/ml) was withdrawn at the age of ten weeks. As previously described, liver samples from control mice and animals with YAP^{S127A} expression were obtained.[2] Tissues or tumors were isolated six weeks (premalignant livers) or 13 weeks (HCC nodules) after transgene induction.

Genetic manipulation of cell lines and drug treatment.

Small interfering RNAs (siRNAs) were transfected using Lipofectamine RNAiMax or Oligofectamine according to the manufacturer’s protocol (Invitrogen/Thermo Fisher Scientific, Darmstadt, Germany). Unless otherwise specified, siRNAs were used at a final concentration of 40 nM. Equimolar 'nonsense' siRNA (siCo) without detectable gene-specificity was used as a negative control. Cells were seeded 24 hours before transfection and harvested as indicated. Sequences of siRNAs are listed in Suppl. Table S2.

For the treatment with the inhibitors Verteporfin (Sigma-Aldrich) and TED-347 (Selleck Chemicals GmbH, Planegg, Germany), cells were incubated with the indicated concentrations. At indicated time points, protein and RNA were isolated for further analysis.

HEK-293T cells were transfected with pMD2.G, psPAX2, and RT3GEPIR vectors for retrovirus production utilizing polyethylenimine (PEI) according to a standard protocol. The virus was collected 48 hours after transfection and used for the infection of A-549 cells. Positive cells were selected utilizing puromycin for two weeks. Inhibition efficiency was tested by Western blotting after administration of DOX (2 mg/ml) for 48 hours.

Western Blotting and fractionation

Western immunoblotting and signal detection were performed as previously described. Signal quantification was carried out using the LiCor system (LiCor Biosciences, Bad Homburg, Germany).

Subcellular protein fractionation was conducted using the NE-PER™ Nuclear and Cytoplasmic Extraction Reagents kit. Cells were seeded at low density (150,000 cells per 10 cm dish). The following day, cells were harvested and processed according to the manufacturer's instructions. The success of fractionation was evaluated by SDS-PAGE and Western blotting. GAPDH and PARP were used as cytoplasmic and nuclear loading controls, respectively.

Viability assay, BrdU enzyme-linked immunosorbent assay (ELISA), and colony formation assay

To measure cell viability, cells were seeded and transfected with gene-specific siRNAs and control siRNA (nonsense) as described. At the indicated time points, Resazurin reagent was added according to the manufacturer's protocol. Light emission was measured using a FLUOstar Omega microplate reader (BMG Labtech, Ortenberg, Germany).

To analyze the effects on proliferation after gene-specific knockdown, BrdU-ELISA assay (Cell proliferation ELISA Biotrak) was performed according to the manufacturer's instructions 72 hours after transfection.

For analyzing colony formation, cells were seeded at low cell numbers (1,000-2,000 cells/well) 24 hours after siRNA transfection into 6-well plates and cultured for 10-14 days. After the cells were washed with PBS, 0.5% crystal violet solution was added, and the cells were incubated for one additional hour. Subsequently, cells were washed with water to remove any crystal violet residues. The colony area was assessed using the ImageJ plugin ColonyArea.[3]

The CYTOR rescue experiment after CYTOR overexpression and YAP and/or TAZ inhibition was performed as previously described.[4]

Quantitative PCR (qPCR) analysis

Total RNA was isolated using the ExtractMe Total RNA Kit according to the manufacturer's instructions (7BioSciences, Neuenburg, Germany). cDNA was synthesized using 500 ng of total RNA with the PrimeScript RT Master Mix following the manufacturer's protocol (TakaraBio, Saint-Germain-en-Laye, France). qPCR reactions were set up using the primaQuant 2x qPCR-SYBR-Green-Mastermix (Steinbrenner Laborsysteme, Wiesenbach, Germany) with the following cycling conditions: 95°C for 15 minutes, followed by 40 cycles of 95°C for 15 seconds and 60°C for 60 seconds (Quant Studio 3 qPCR system; Applied Biosystems, Darmstadt, Germany). Subsequent melting curve analysis was applied to ensure product specificity (95°C for 15 seconds, 60°C for 30 seconds, 60-95°C with 0.1°C/second). The housekeeping genes GAPDH, RPL41, or SRSF4 were used to normalize samples from human cells. The housekeeping genes Actb, Gapdh, Hprt, Ppia, or Tubb5 were used to normalize murine samples. Primers are listed in Suppl. Table S2. The murine gene Morrbid is regarded as the orthologue of the human genes CYTOR and MIR4435-2HG (https://www.ncbi.nlm.nih.gov/gene/100043424).

Chromatin immunoprecipitation (ChIP)

ChIP assay was performed as previously described.[2] In brief, HLF cells were seeded onto 15 cm dishes and incubated until reaching about 80% confluency. Cells were fixed with 1%

formaldehyde in PBS for 15 minutes and quenched with 2.5 M glycine for 5 minutes. Subsequently, cells were harvested in RIPA buffer supplemented with 1x Protease Inhibitor Mix G and sonicated to generate DNA fragments of less than 500 bp. After preclearing, samples were mixed with 2 µg of specific antibody or IgG as control and blocked Dynabeads followed by incubation at 4°C overnight. After several washing steps (4 x RIPA, 4 x IP wash buffer, 2 x TE), the protein-DNA complexes were eluted from the Dynabeads. Cross-linking was reversed by adding 4 M NaCl and incubation at 65°C for five hours. DNA was purified using the NucleoSpin® Gel and PCR Clean-up Kit according to the manufacturer's instructions. Finally, precipitated DNA was quantified with qPCR using a serial dilution of genomic DNA to calculate a reference standard curve. ChIP primers were designed based on the TEAD4 binding sites identified by ChIP-Seq data analysis and the prediction of TEAD4 binding sites using the JASPAR database.[5] Commercially available primers covering the human CTGF promoter and primers covering the CTGF upstream region without transcription factor binding site were employed as positive and negative controls, respectively (SimpleChIP®, Cell Signaling). Primers are listed in Suppl. Table S2.

Immunofluorescence

For immunofluorescence stains, HLF cells were seeded on coverslips under different cell density conditions ranging from low (100,000 cells in 6 well plates) to high density (500,000 cells in 6 well plates). After 24 hours, cells were fixed using 4% paraformaldehyde in PBS for 15 minutes, followed by permeabilization in 0.2% Triton X-100/PBS for 15 minutes. Cells were blocked with 1% BSA in PBS for 30 minutes and incubated with the primary antibody for one hour (1:60). The next day, cells were treated with the secondary antibody for one hour (1:200). Subsequently, the coverslips were dried and mounted on microscopy slides with DAPI Fluoromont-G®. Images were acquired at 40x magnification with an Olympus IX81 microscope using the Olympus CellSens Dimension software (Olympus, Shinjuku, Japan). Image processing was performed using ImageJ (v1.53f51).[6] Antibodies used in this manuscript are listed in Suppl. Table S3.

Immunohistochemistry and image analysis

Immunohistochemical staining was performed on FFPE tissue sections. In brief, slides were deparaffinized and rehydrated using xylene (3 x 5 minutes), 100% ethanol (2 x 2 minutes), 96% ethanol (2 x 2 minutes), 70% ethanol (2 x 2 minutes), and rinsed with aqua dest. Antigen retrieval was performed in a pressure cooker (YAP and TAZ, 8 minutes; Ki-67, 15 minutes) with Target Retrieval Solution Citrate pH 6, respectively. Slides were washed with TBS (YAP, Ki-67) for 10 minutes and subsequently incubated with a primary antibody at 4°C overnight (YAP, TAZ) or for one hour at room temperature (Ki-67). Slides were washed twice with TBS for 5 minutes and incubated with the secondary antibody for 45 minutes. After two additional washing steps with TBS, the signal was detected by the Permanent AP Red Kit for 5 minutes. According to the manufacturer's protocol, tissue slides from cohort 2 were stained using a

Mouse and Rabbit Specific HRP/DAB IHC Detection Kit (Abcam, Cambridge, UK). Antibodies used in this manuscript are listed in Suppl. Table S3.

For a visual stain quantification, a score was derived from the following scoring system: quantity (1, ≤1% positive; 2, 1%–5% positive; 3, 6%–20%; and 4, ≥20% positive cells) and intensity (1, low/not detected; 2, moderate; and 3, high). The product of quantity and intensity was calculated (range: 1-12). For YAP, nuclear and cytoplasmic staining scores were evaluated separately. For Ki-67 stains, only quantity scores were determined (range 1-4). Regarding *in situ* hybridization analysis of TMAs, a scoring system consisting of quantitative parameters was applied (1, not detected; 2, very low; 3, low; 4, moderate; and 5, high). Two experienced investigators performed visual evaluations.

To quantify *in situ* hybridization of the lncRNA Morrbid, stained mouse liver tissue sections were digitalized using digital slide scanners (Aperio AT2, Leica Mikrosysteme Vertrieb GmbH, Wetzlar, Germany) at 40x magnification. The digital whole slide images of mouse liver tissue samples were divided into tiles (1 mm^2 size) using OpenSlide bindings to Python via open-source Automated Slide Analysis Platform (ASAP) software (https://computationalpathologygroup.github.io/ASAP/). All tiles were used for image processing and quantification, except those with staining or scanning artifacts and tissue areas with less than 50% tissue content. A random forest machine learning algorithm was trained to recognize hybridized and stained Morrbid within the mouse liver tissue slides using Ilastik software (v1.3.3).[7] The trained algorithm was applied to all tiles afterward to generate probability maps of the detected classes. The resulting probability maps were thresholded and the counts of Morrbid signals were quantified per tissue area with ImageJ scripts (v1.53q).[8]

In situ hybridization and dual staining

Detection of RNA molecules on formalin-fixed, paraffin-embedded (FFPE) tissue slides was performed using an RNAscope 2.5HD kit "brown" according to the manufacturer's protocol (Advanced Cell Diagnostics, Bio-Techne, Minneapolis, USA). In brief, tissue samples were cut into 5 µm sections and heated at 60°C for one hour. Sections were deparaffinized by washing slides with xylene (2 x 5 minutes) and 100% ethanol (2 x 1 minute). Tissues were dried and hydrogen peroxide was added at RT for 10 minutes. The slides were treated with boiling target retrieval solution for 15 minutes and dried at RT overnight. The next day, protease plus was applied, and the slides were incubated at 40°C for 45 minutes in a humidified oven. Hybridization with specific probes was performed at 40°C for 2.5 hours. Afterwards, the signal was amplified in 6 consecutive steps. The signal was detected by incubating the slides with 3,3′-diaminobenzidine (DAB) at room temperature for 10 minutes. For counterstaining, the slides were incubated in 50% hematoxylin for 1 minute and washed in 0.02% ammonia water for 10 seconds. Probes for CYTOR, SNHG1, and Morrbid were designed and synthesized by the manufacturer. PPIB and DapB genes served as positive and negative controls, respectively.

For the dual staining, the RNAscope 2.5HD kit "red" was used according to the manufacturer's protocol (Advanced Cell Diagnostics, Bio-Techne). After antigen retrieval, wash with water twice for 1 minute each, then wash with TBS-Tween once for 1 minute, followed by incubation

with the primary antibody overnight at 4°C. After several washing steps with TBS-Tween (4 x 1 minute), protease plus was applied, and the slides were incubated at 40°C. Hybridization with specific probes was performed at 40°C for 2.5 hours. Afterward, the signal was amplified in 6 consecutive steps. After adding Co-detection blocking buffer, slides were washed twice with 1x wash buffer for 2 minutes and incubated with POLYVIEW® Plus HRP antibody for 1 hour.

Supplementary Table S1

Nr.	Age	Gender	Time between blood collection and tissue collection (TC) (Months; +: after TC, -: before TC)	Cancer diagnosis	Grading	Staging	Cohort
cohort1_1	75	m	+136	HCC	NA	NA	cohort1
cohort1_2	44	m	+3	HCC	G3	pT1a, Nx	cohort1
cohort1_3	49	m	+18	HCC	G2	NA	cohort1
cohort1_4	50	m	+23	HCC	G1	pT1, Nx	cohort1
cohort1_5	60	f	+19	HCC	G2	pT1, N1	cohort1
cohort1_6	72	m	+12	HCC	G1	NA	cohort1
cohort1_7	60	m	+11	HCC	G2	pT1, N0	cohort1
cohort1_8	62	m	-1	HCC	G3	pT2, N0, Mx	cohort1
cohort1_9	69	m	-0,5	HCC	G2	pT1, NX	cohort1
cohort1_10	65	f	-0,5	HCC	G2	pT1, Nx	cohort1
cohort1_11	61	f	+42	HCC	G2	pT2, Nx	cohort1
cohort1_12	61	m	+2	HCC	G2	pT3a, Nx	cohort1
cohort1_13	65	m	+3	HCC	G2	pT1, Nx	cohort1
cohort1_14	70	m	+5	HCC	G3	pT1, NX	cohort1
cohort1_15	75	f	0	HCC	G2	pT2, Nx, Mx	cohort1
cohort1_16	69	m	+16	HCC	G2	pT3a, N0	cohort1
cohort1_17	74	m	+57	HCC	G2	pT2, Nx, pMx	cohort1
cohort2_1	55	m	0	HCC	G2	pT2, N0, M0 (stage II)	cohort2
cohort2_2	60	m	0	HCC	G2	pT2, N0, M0 (stage II)	cohort2
cohort2_3	57	m	0	HCC	G1	pT1b, N0, M0 (stage IB)	cohort2
cohort2_4	59	m	0	HCC	G2	pT1a, N0, M0 (stage IA)	cohort2
cohort2_5	74	m	0	HCC	G2	pT2, N0, M0 (stage II)	cohort2
cohort2_6	53	f	0	HCC	G2+3	pT4, N0, M0	cohort2
cohort2_7	55	m	0	HCC	G2	pT2, N0, M0 (stage II)	cohort2
cohort2_8	66	m	0	HCC	G2	pT2, N0, M0 (stage II)	cohort2

Supplementary Table 2
Oligos used in the manuscript

Oligos were ordered from Thermo Fisher Scientific
Human SNHG17 ChIP forward primer: GTTACCCGCTGTGCATCTCT
Human SNHG17 ChIP reverse primer: AATGAATTCTACCCCCGCCC
Human MIR4435-2HG ChIP forward primer: AGACCTACCGGAAGGATCAGA
Human MIR4435-2HG ChIP reverse primer: ACTGGAAAAATGTAGGTTGCACG
Human DLEU1 ChIP forward primer: GAAAGCGCTGAGGCTCCTC
Human DLEU1 ChIP reverse primer: GTCGGGAAAGCAAGAGAAGGG
Human DLEU1 ChIP forward primer control: TATTTGCAACCCAGAGCCGT
Human DLEU1 ChIP reverse primer control: CTTTGCCGGTGTATTGCGTG
Human FTX ChIP forward primer: TGTGTTTCCAAATGCTTGTTAC
Human FTX ChIP reverse primer: CATACCACCACTGCCATTAACTACTT
Human FTX ChIP forward primer control: AGGTCAGACAAGGCTGGGTAAG
Human FTX ChIP reverse primer control: GAGTGGAGGTCTTGCCCAGG
Human CYTOR forward primer: ATGCCCAAAGTTACGGAGGA
Human CYTOR reverse primer: TATTCGAGGGATGCAGACGG
Human SNHG1 forward primer: ACGTTGGAACCGAAGAGAGC
Human SNHG1 reverse primer: GCAGCTGAATTCCCCAGGAT
Human MIR4435-2HG forward primer: GTCATTAAGGTGGTCCTGCC
Human MIR4435-2HG reverse primer: AGTGTCCTTTTCAGCGAGTGA
Human SNHG17 forward primer: AGCGTAGCTTCCTTGTCGTG
Human SNHG17 reverse primer: GAGACCTGACAGACAGCGTG
Human DLEU1 forward primer: TTACCAGATGAGGACACCTGAG
Human DLEU1 reverse primer: AAGAATGGCTGGCAAAGGCT
Human FTX forward primer: TCCTGTGCCTGCTGTCCATT
Human FTX reverse primer: TGTGGCATCACCTCCTGGTT
Human ANKRD1 forward primer: AGTAGAGGAACTGGTCACTGG
Human ANKRD1 reverse primer: TGGGCTAGAAGTGTCTTCAGAT
Human CTGF forward primer: CCAAGGACCAAACCGTGG

Human CTGF reverse primer: CTGCAGGAGGCGTTGTCAT
Human CYR61 forward primer: AGCCTCGCATCCTATACAACC
Human CYR61 reverse primer: TTCTTTCACAAGGCGGCACTC
Human YAP1 forward primer: CCTGCGTAGCCAGTTACCAA
Human YAP1 reverse primer: CCATCTCATCCACACTGTTC
Human WWTR1 forward primer: CAGAGAATCCAGATGGAGAG
Human WWTR1 reverse primer: GTTGACAGCAGCCTGAACTG
Human TEAD1 forward primer: GACAGTCACCTGTTCCACCAAAG
Human TEAD1 reverse primer: CCATTCTCAAACCTTGCATACTCCG
Human TEAD2 forward primer: CTCACCTGTTCCTCCAAGGTC
Human TEAD2 reverse primer: CACCAGGTACTCGCACATGG
Human TEAD3 forward primer: TTCATGGAGGTGCAGCGAGAC
Human TEAD3 reverse primer: CGCACATCTACTGCCTCCAG
Human TEAD4 forward primer: TGGAGTTCTCTGCCTTCCTG
Human TEAD4 reverse primer: GGACTGGCCAATGTGCACGA
Human GAPDH forward primer: CTGGTAAAGTGGATATTGTTGCCAT
Human GAPDH reverse primer: TGGAATCATATTGGAACATGTAAACC
Human RPL41 forward primer: AAACCTCTGCGCCATGAGAG
Human RPL41 reverse primer: AGCGTCTGGCATTCCATGTT
Human SRSF4 forward primer: TGCAGCTGGCAAGACCTAAA
Human SRSF4 reverse primer: TTTTTGCGTCCCTTGTGAGC
Custom Spike-In Oligo: TGCTGTTGACAGTGAGCGCCCGCTGCATTTATAAAGAA TATAGTGAAGCCACAGATGTATATTCTTTATAAATGCAG CGGATGCCTACTGCCTCGGA
Spike-In forward primer: GAGCGCCCGCTGCATTTA
Spike-In reverse primer: GTAGGCATCCGCTGCATTTA
Murine Actb forward primer: GCTTCTTTGCAGCTCCTTCGT
Murine Actb reverse primer: ACCAGCGCAGCGATATCG
Murine Gapdh forward primer: TGTCCGTCGTGGATCTGAC
Murine Gapdh reverse primer: CCTGCTTCACCACCTTCTTG

Murine Hprt forward primer: TCCTCCTCAGACCGCTTTT
Murine Hprt reverse primer: CCTGGTTCATCATCGCTAATC
Murine Ppia forward primer: GCATACAGGTCCTGGCATCT
Murine Ppia reverse primer: AGCTGTCCACAGTCGGAAAT
Murine Tubb5 forward primer: TCACTGTGCCTGAACTTACC
Murine Tubb5 reverse primer: GGAACATAGCCGTAAACTGC
Murine Snhg1 forward primer: GCTTGTAGTCAGGGTGCTGT
Murine Snhg1 reverse primer: AAACCTGCACTCATCCTGGG
Murine Morrbid forward primer: CAAAGCAAACCAGAGGACCAG
Murine Morrbid reverse primer: TCAACCCAACAGGTTGTCATCA
Murine Snhg17 forward primer: TGAAGGTGAGCCACTTCGGA
Murine Snhg17 reverse primer: AGCGACACGTTACTTCCTCTG

Commercial available oligos (Cell Signaling Technology)
SimpleChIP® Human CTGF Promoter Primers #14927
SimpleChIP® Human CTGF Upstream Primers #14928

Oligos ordered from Microsynth Seqlab GmbH	
siYAP1#1	CCA CCA AGC UAG AUA AAG A dTdT
siYAP1#2	GGU CAG AGA UAC UUC UUA A dTdT
siTAZ#2	AAA CGU UGA CUU AGG AAC UUU dTdT
siTAZ#3	AGG UAC UUC CUC AAU CAC A dTdT
siCo	UGG UUU ACA UGU CGA CUA A dTdT
siTEAD#1 (15 nucleic acids overlap with siTEAD#2)	AUG AUC AAC UUC AUC CAC A dTdT
siTEAD#2 (15 nucleic acids overlap with siTEAD#1)	UCA ACU UCA UCC ACA AGC U dTdT
siCYTOR#1	CAG UCU CUA UGU GUC UUA A dTdT
siCYTOR#2	CAC ACU UGA UCG AAU AUG A dTdT
siSNHG1#1	GAG CAA AUA AGG UGU AUA AAA dTdT
siSNHG1#2	CAG CUU GUU GCU AUA UAC CAU dTdT
siSNHG17#1	UUA CCC ACC CAU UCA AUA A dTdT
siSNHG17#2	GGU GAC GUG UGU UCA AGA A dTdT
siMIR4435-2HG#1	GGC ACA AUU UAA UCC AUA A dTdT
siMIR4435-2HG#2	GGA UCA CCG CUA AAG AAA A dTdT

Supplementary Table 3
antibodies used in the manuscript

Anti-YAP (D8H1X) XP	Cell Signaling Technology	Cat# 14074, RRID: AB_2650491
Anti-TAZ (E9J5A) XP	Cell Signaling Technology	Cat# 72804, RRID: AB_2904134
Pan-TEAD (D3F7L)	Cell Signaling Technology	Cat# 13295, RRID: AB_2687902
Anti-GAPDH	Millipore	Cat# AB2302, RRID: AB_10615768
Anti-PARP	Cell Signaling Technology	Cat# 9542, RRID: AB_2160739
Rabbit serum (normal)	Agilent	Cat# X0902
Anti-Ki67	Abcam	Cat# ab15580, RRID:AB_443209
Alexa fluor 488 donkey anti-rabbit IgG	Jackson Immuno Research	Cat# 711-545-152, RRID:AB_2313584
Polyview plus HRP anti-rabbit	Enzo	Cat# ENZ-ACC103-0150

Supplementary Table S4

RNA sequencing of HLF cells (GSE207724 subseries GSE207722)

SampleID	Total_number of_sequenced_ reads	Total_number_of_ uniquely_mapped_ reads	RIN	Total_number_o f_pseudoaligned _reads	Percent_Pseudoaligned_ Reads	Perscent_Unique _Reads
YAP2TAZ3_1Y2T3	41127334	11681609	>7	24797536	60.3	28.4
YAP2TAZ3_2Y2T3	40929821	14701835	>7	31642662	77.3	35.9
YAP2TAZ3_3Y2T3	41529925	14657653	>7	31192084	75.1	35.3
YAP2TAZ3_4Y2T3	41879414	15392389	>7	32757333	78.2	36.8
YAP1TAZ2_1Y1T2	41763941	11162812	>7	23634773	56.6	26.7
YAP1TAZ2_2Y1T2	41652019	14744994	>7	31466722	75.5	35.4
YAP1TAZ2_3Y1T2	24616996	6766279	>7	14211685	57.7	27.5
YAP1TAZ2_4Y1T2	40779941	14698062	>7	31340834	76.9	36
Nonsense_1NTC	42182101	13031363	>7	27695707	65.7	30.9
Nonsense_2NTC	41286131	13182885	>7	27982069	67.8	31.9
Nonsense_3NTC	41451247	15247869	>7	32601716	78.7	36.8
Nonsense_4NTC	41390918	14860845	>7	31650945	76.5	35.9

RNA sequencing of A549 cells (GSE207724 subseries GSE207723)

SampleID	Total_number_ of_sequenced_ reads	Total_number_of_ uniquely_mapped_ reads	RIN	Total_number_o f_pseudoaligned _reads	Percent_Pseudoaligned_ Reads	Perscent_Unique _Reads
NTC1	56223313	9703146	>7	35185313	62.6	17.3
Y1T21	55409508	9112118	>7	33381761	60.2	16.4
Y2T31	56722623	9184514	>7	32818928	57.9	16.2
NTC2	58591697	9820438	>7	35402622	60.4	16.8
Y1T22	58379760	9681664	>7	35525147	60.9	16.6
Y2T32	48635768	8391707	>7	30560364	62.8	17.3
NTC3	50603275	8502121	>7	30092838	59.5	16.8
Y1T23	62771830	10599853	>7	38284875	61	16.9
Y2T33	53626773	9484666	>7	35650700	66.5	17.7
NTC4	58270311	10108232	>7	37289042	64	17.3
Y1T24	49513876	8637435	>7	33387020	67.4	17.4
Y2T34	43132339	7781973	>7	29631949	68.7	18

Supplementary Table 5: Correlation of four lncRNA signature with the YAP-dependent mRNA signature #2 in 32 tumor types (TCGA database for which lncRNA data is available)

	Tumor type	correlation (r)	p-value	patients (n)
high correlation	**NSCLC (LUAD)**	0.5225	≤0.001	510
	ACC	0.4958	≤0.001	79
	STAD	0.4682	≤0.001	373
	LGG	0.3675	≤0.001	500
	MESO	0.364	≤0.001	81
	TGCT	0.3245	≤0.001	149
	READ	0.3189	≤0.001	163
	KIRC	0.3021	≤0.001	526
	KIRP	0.2955	≤0.001	287
	BRCA	0.2894	≤0.001	1072
	**LIHC (HCC)**	0.2863	≤0.001	369
	COAD	0.2825	≤0.001	453
	UVM	0.2674	≤0.02	77
moderate correlation	GBM	0.2479	0.003	144
	THYM	0.2459	0.007	119
	BLCA	0.2395	≤0.001	405
	PRAD	0.2206	≤0.001	481
	HNSC	0.2171	≤0.001	495
	LUSC	0.2157	≤0.001	496
	SKCM	0.2122	0.03	103
	THCA	0.21	≤0.001	497
no/weak correlation	CHOL	0.183	0.29	36
	UCEC	0.1783	≤0.001	537
	PAAD	0.171	0.02	177
	ESCA	0.1593	0.05	152
	OV	0.1562	0.003	354
	SARC	0.1457	0.02	258
	UCS	0.1412	0.47	56
	CESC	0.1151	0.05	296
	DLBC	0.1014	0.5	47
	PCPG	0.0893	0.11	175
	KICH	0.043	0.73	65

Suppl. Figure S1

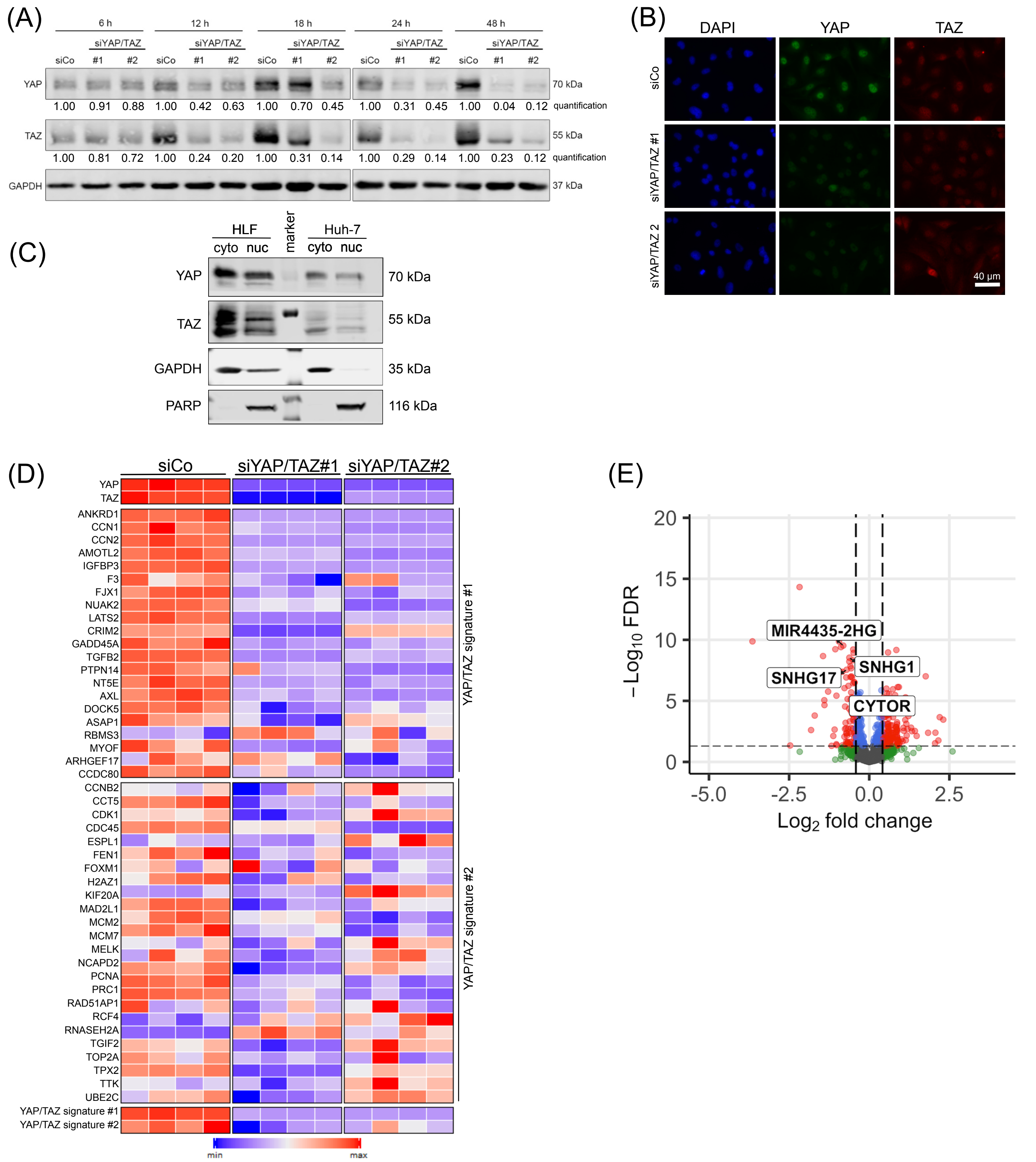

Supplementary Figure S1: Identification of YAP/TAZ-regulated lncRNAs in HCC cells

(A) Western immunoblot demonstrates effective inhibition of YAP and TAZ proteins in HLF cells following transfection with two siRNA combinations at the specified time points (siYAP/TAZ #1 and #2). Inhibition after 24 hours was chosen for NGS analysis due to the efficient reduction of both proteins at the earliest time. The numbers indicate the relative decrease in protein intensity compared to the respective control (siCO).

(B) Immunofluorescence staining of endogenous YAP and TAZ was performed after transfection with siYAP/TAZ #1 and siYAP/TAZ #2. Images were captured 48 hours post-transfection.

(C) Western immunoblot after fractionation of cytoplasmic and nuclear proteins from HLF and Huh-7 cells. GAPDH and PARP (poly[ADP-ribose]-polymerase 1) serve as fractionation controls.

(D) Heatmap summarizing NGS data for target genes in HLF cells following 24 hours of YAP/TAZ inhibition. Two published YAP/TAZ target gene signatures were utilized, with 21/22 genes and 25/25 genes detectable in the NGS data.[18, 30] The equally weighted average score of genes is shown again at the bottom of the heatmap. Two combinations of siRNAs targeting YAP and TAZ were utilized (#1, #2). Four biologically independent samples were analyzed for each inhibition and control.

(E) Exemplary volcano plot of differentially expressed lncRNAs following YAP/TAZ siRNA#1 inhibition in HLF cells. Signature lncRNAs are highlighted. Horizontal dashed lines indicate FDR=0.05, while vertical dashed lines indicate FC=±0.75.

Suppl. Figure 2

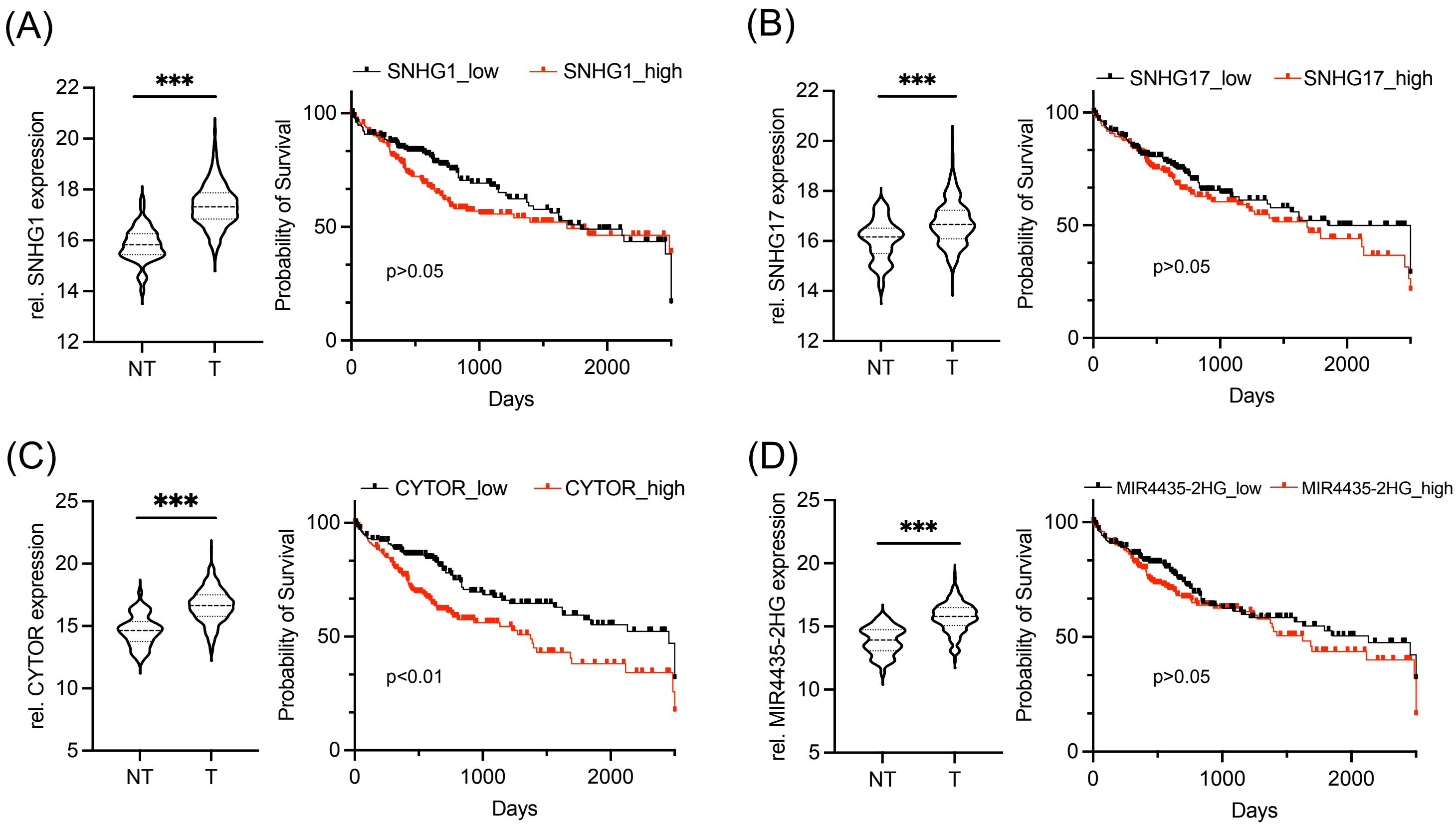

Supplementary Figure S2: Expression of lncRNAs in HCC patients

The violin plots and Kaplan-Meier curves of **(A)** SNHG1, **(B)** SNHG17**, (C)** CYTOR, and **(D)** MIR4435-2HG using TCGA data from HCC patients. Statistical test for violin plots: Mann–Whitney U test, ***$p \leq 0.001$. Non-tumor tissues (NT) and tumor tissues (T). The division into "low" and "high" expression groups for the Kaplan-Meier survival analysis was based on the average expression values of the respective lncRNA. Statistical comparisons were performed using the log-rank (Mantel-Cox) test.

Suppl. Figure S3

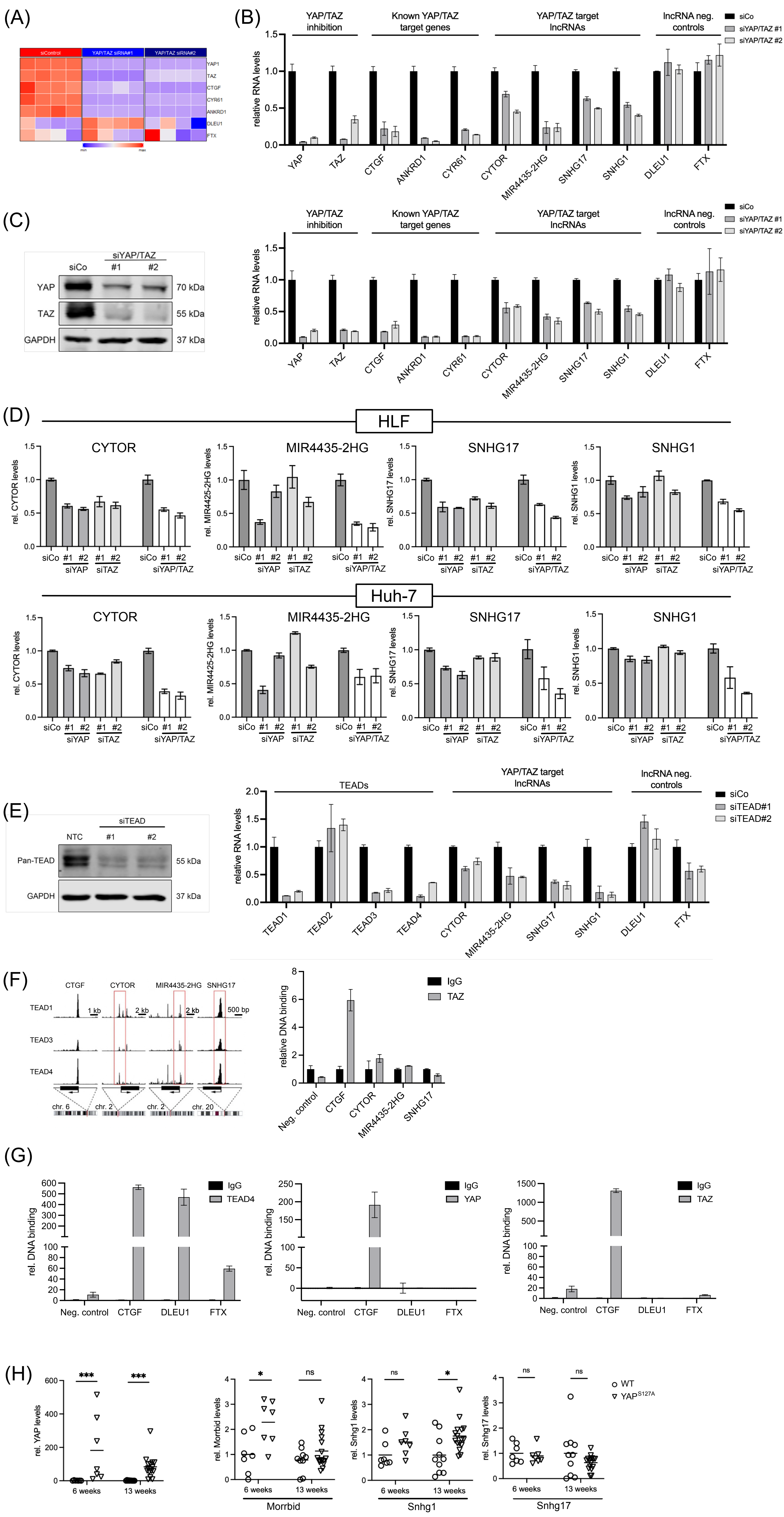

Supplementary Figure S3: Mechanism of YAP/TAZ-dependent lncRNA expression in cancer cells

(A) Heatmap summarizing NGS data for YAP and TAZ, positive controls (CTGF, CYR61, ANKRD1), and lncRNAs DLEU1 and FTX in HLF cells following YAP/TAZ silencing. DLEU1 and FTX were chosen as negative controls for all confirmatory experiments since no significant regulation was observed.

(B) Confirmatory qPCR analysis of YAP/TAZ, its target genes (CTGF, ANKRD1, CYR61), lncRNA signature constituents (CYTOR, MIR4435-2HG, SNHG17, SNHG1), and negative controls (DLEU, FTX) in HLF cells after combined YAP/TAZ inhibition for 24 hours.

(C) Western immunoblot analysis of YAP and TAZ demonstrates significant protein reduction following the transfection of specific siRNAs in Huh-7 cells. The qPCR data for YAP/TAZ, known YAP/TAZ target genes, lncRNA signature constituents, and negative control lncRNAs after 24 hours of combined YAP/TAZ inhibition are presented.

(D) Inhibition of YAP or TAZ alone and in combination using two different gene-specific siRNAs in HLF and Huh-7 cells (20 nM for single and 40 nM for combined inhibition). LncRNA levels were measured 48 hours after transfection.

(E) Western immunoblot for all TEAD isoforms following transfection of siRNAs (#1, #2) targeting TEAD1, TEAD3, and TEAD4 in HLF cells. Please note that both siRNAs share an identical 15-nucleotide region due to structural constraints and therefore cannot be considered independent. No other siRNAs targeting TEAD1/3/4 family members could be designed. The qPCR analysis of all TEAD family members, signature lncRNAs, and negative control lncRNAs in HLF cells after 24 hours is shown.

(F) Outline of TEAD1/3/4 ChIP-seq profiles in the promoter regions of lncRNA candidates (CYTOR, MIR4435-2HG, SNHG17) and the positive control CTGF. The red box highlights the genomic area utilized for primer design. Binding sites in the promoters of lncRNAs were selected using the JASPAR database. ChIP experiments were conducted for CYTOR, MIR4435-2HG, and SNHG17 with TAZ in HLF cells.

(G) ChIP analysis of TEAD, YAP, and TAZ binding to the promoters of the negative control genes DLEU1 and FTX in HLF cells. One of the two representative experiments is shown.

(H) qPCR analysis of YAP and lncRNAs for which mouse orthologues exist (Morrbid, Snhg1, Snhg17). Premalignant liver tissues from wildtype (WT, n=7) and YAP^{S127A}-transgenic (n=7) mice were examined after withdrawing doxycycline for six weeks. Liver cancer nodules from WT (n=10) and YAP^{S127A}-transgenic mice (n=16) were isolated 13 weeks after transgene induction.

Suppl. Figure S4

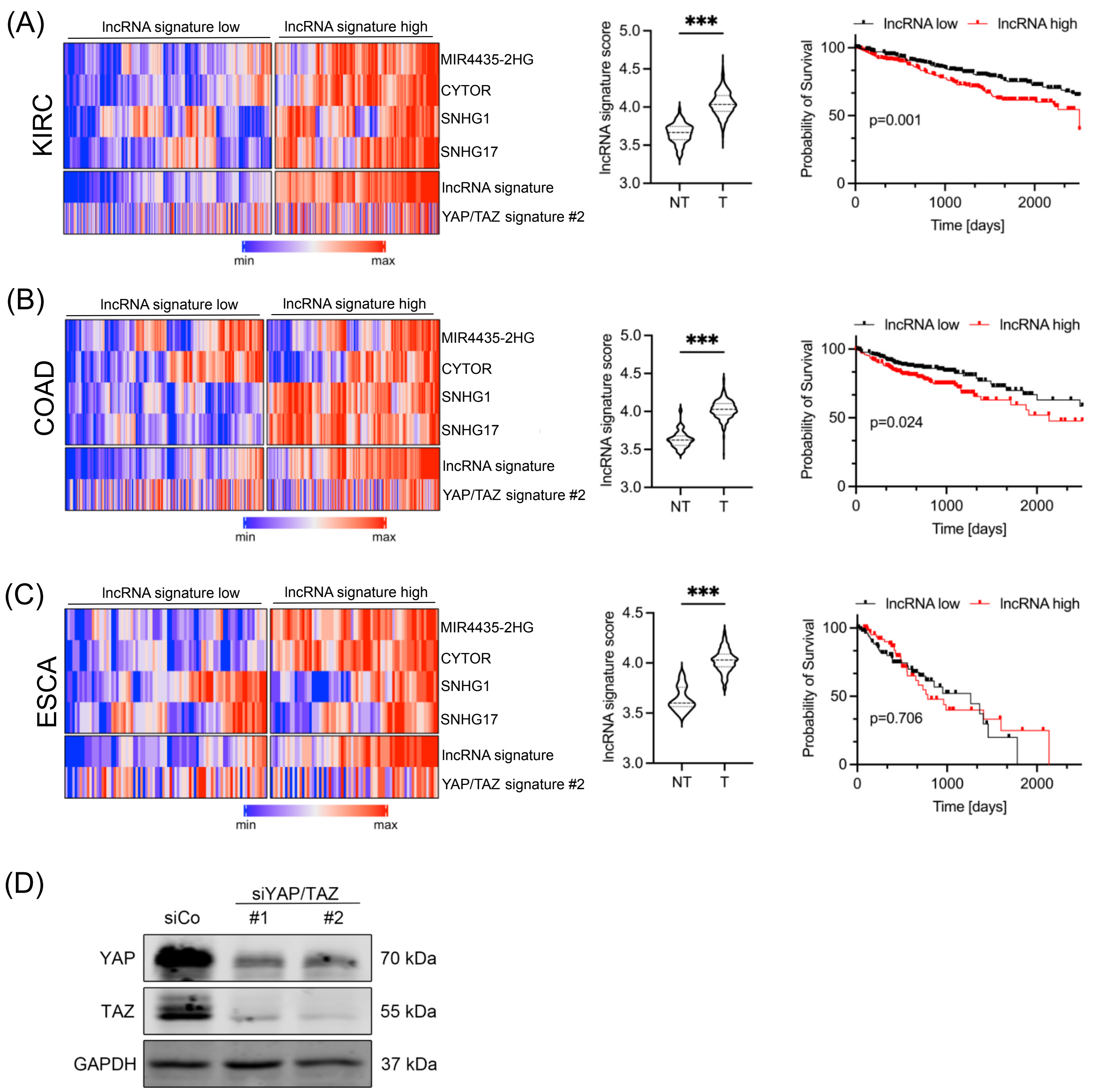

Supplementary Figure S4: Detection of YAP/TAZ-regulated lncRNAs in other cancer types

The presence of the four lncRNA signature was analyzed across 32 tumor types. For example, in kidney renal clear cell carcinoma (KIRC; **A**) and colon adenocarcinoma (COAD; **B**), a stronger association was found between the lncRNA and the YAP target gene signature.

(C) In esophageal carcinoma (ESCA), a weak correlation exists between lncRNA and YAP target gene signatures. The respective heatmap after K-mean clustering, expression of the lncRNA signature in non-tumor tissues (NT) tumor tissue (T) specimen, as well as patient survival curve are shown. The p-value is indicated (Statistical tests: log-rank and Gehan-Breslow-Wilcoxon).

(D) Western immunoblot demonstrates inhibition of YAP and TAZ proteins in A-549 cells following transfection with two siRNA combinations at the specified time points (siYAP/TAZ #1 and #2) after 24 hours.

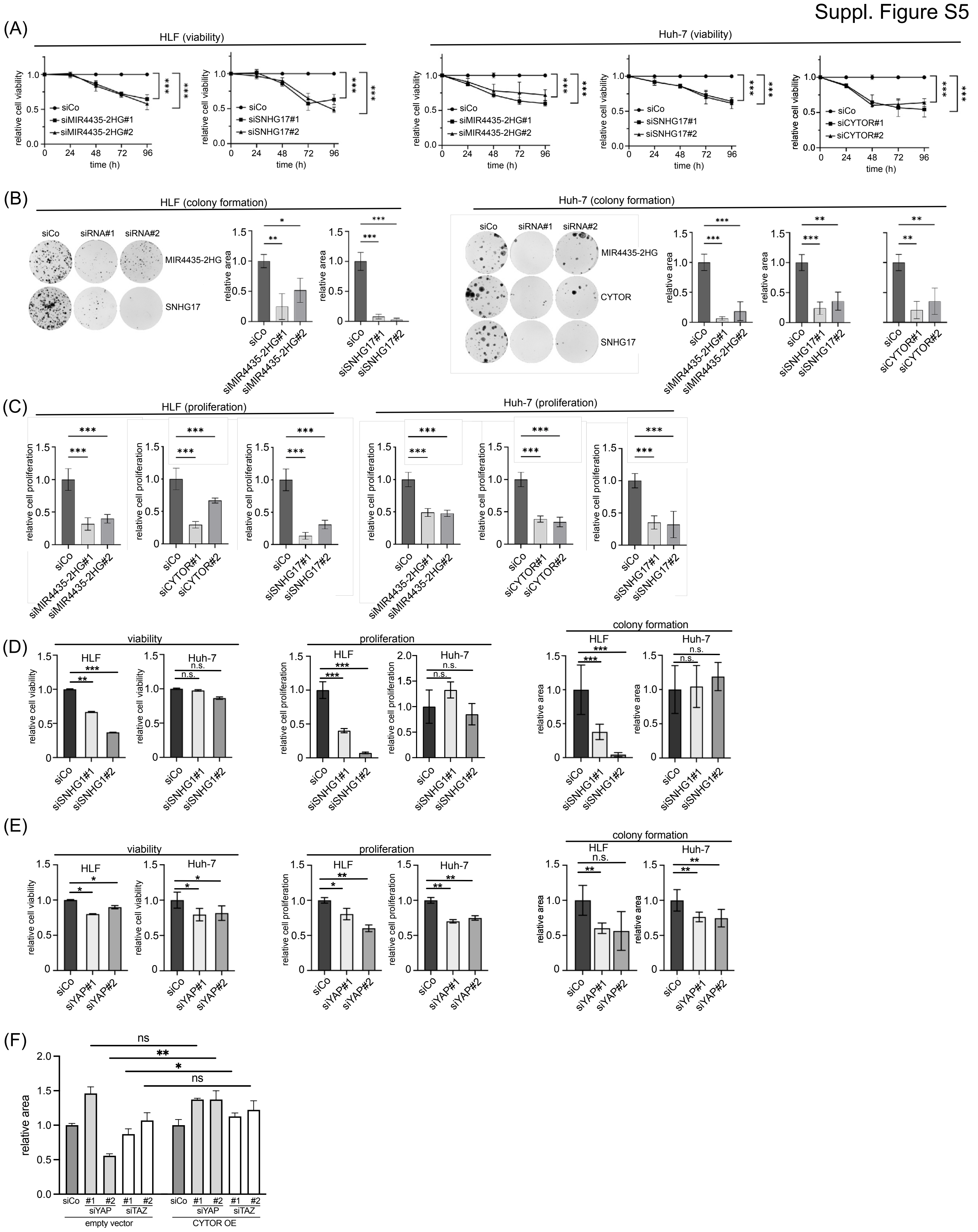

Supplementary Figure S5: Pro-tumorigenic function of YAP/TAZ-induced lncRNAs

(A) Resazurin-based cell viability assays were conducted following the siRNA-mediated inhibition of MIR4435-2HG and SNHG17 in HLF cells, as well as MIR4435-2HG, SNHG17, and CYTOR in Huh-7 cells.

(B) Colony formation assays were conducted following siRNA-mediated inhibition of MIR4435-2HG and SNHG17 in HLF cells, as well as MIR4435-2HG, SNHG17, and CYTOR in Huh-7 cells

(C) Proliferation assays (BrdU ELISA) were carried out after siRNA-mediated inhibition of MIR4435-2HG, SNHG17, and CYTOR in HLF and Huh-7 cells.

(D) Analysis of the functional relevance of SNHG1 in HLF and Huh-7 cells. Note that SNHG1 knockdown is not effective in Huh-7 cells, whereas its inhibition significantly reduces viability, proliferation, and colony formation in HLF cells.

(E) Reduction of viability, proliferation, and colony formation in HLF and Huh-7 cells upon YAP inhibition (positive control).

(F) Colony formation assay was performed using HLF cells with stable overexpression of CYTOR following siRNA-mediated knockdown of YAP or TAZ, respectively. HLF cells stably transfected with an empty vector served as a control. OE: overexpression.

Graphs summarize the results of independent experiments/biological replicates. siCo-transfected cells were utilized as controls, and the results were normalized to respective controls. Each experiment used two different siRNAs (#1, #2). Statistical test: Dunnett's multiple comparison test, *$p \leq 0.05$, **$p \leq 0.01$, ***$p \leq 0.001$.

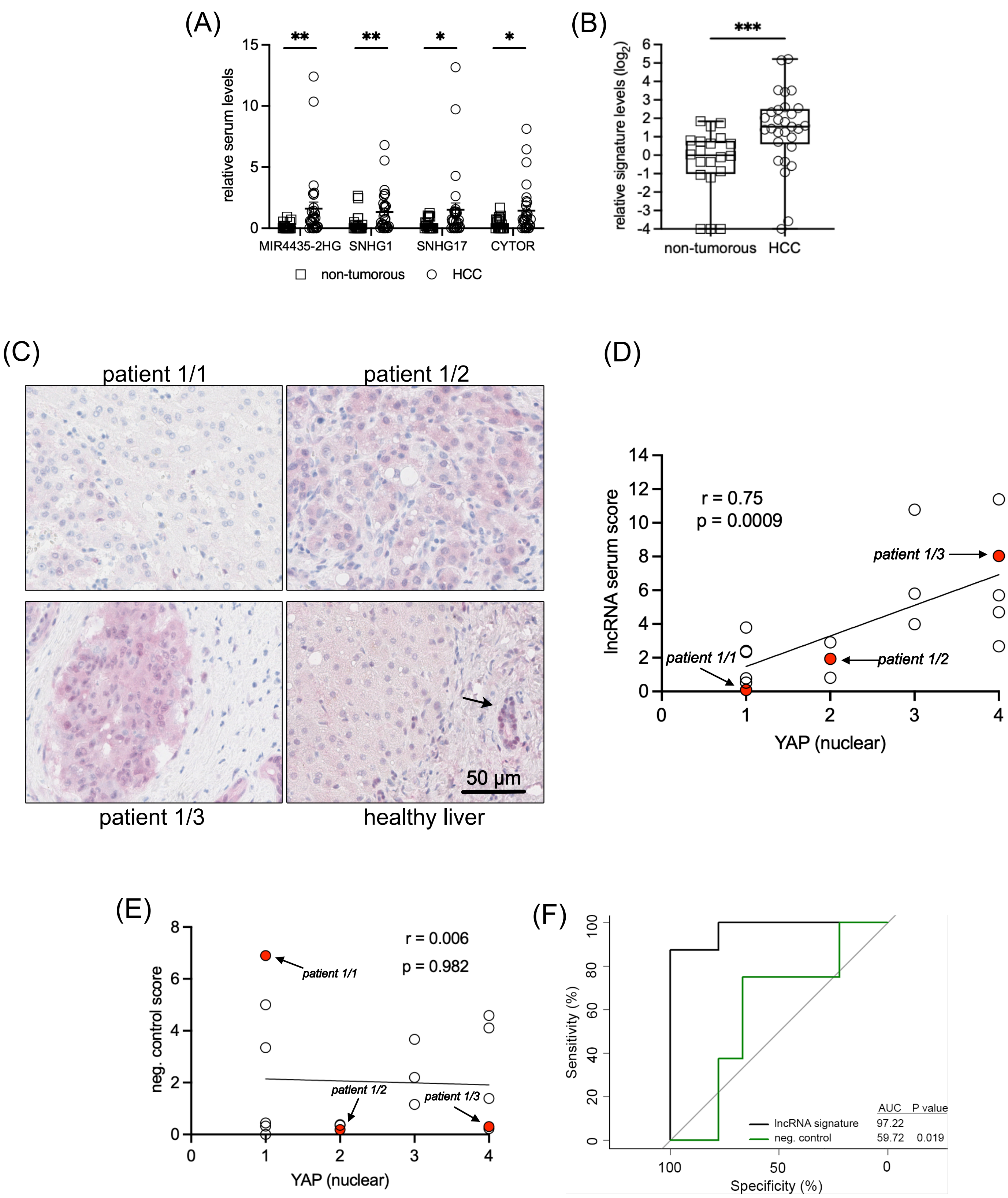

Supplementary Figure S6: Signature lncRNAs in serum correlate with YAP expression in HCC cells

(A) qPCR analysis of signature lncRNAs in the serum of healthy persons (n=20) and HCC patients (n=29) (cohort 1). Statistical test: Mann-Whitney U. ***p≤0.001

B) Boxplot illustrating the balanced lncRNA signature score in serum derived from healthy persons and HCC patients (cohort 1). Statistical test: Mann-Whitney U. ***p≤0.001

(C) Representative YAP stains of cohort 1 HCC patients with low (patient 1/1), intermediate (patient 1/2), and high nuclear YAP abundance (patient 1/3). Normal liver tissue with positively stained cholangiocytes (arrow) was used as a control for the staining protocol.

(D) The graph illustrates the association between lncRNA signature levels in serum and nuclear YAP abundance in corresponding serum/tissue specimens. Line: linear regression. Patients 1/1, 1/2, and 1/3 are highlighted. Statistical test: Spearman correlation. The p-value is indicated.

(E) Linear regression analysis was performed for negative control lncRNAs (DLEU1, FTX) in serum and nuclear YAP abundance in corresponding serum/tissue specimens (cohort 1). Patients 1/1, 1/2, and 1/3 are indicated. Line: linear regression. Statistical test: Spearman correlation. The p-value is indicated.

(F) ROC curve analysis comparing the performance of the lncRNA signature and the negative control lncRNA signature (DLEU1, FTX) in HCC patients. Statistical test: Spearman correlation. The p-value is indicated.

References

1. Camargo FD, Gokhale S, Johnnidis JB, Fu D, Bell GW, Jaenisch R, Brummelkamp TR. YAP1 increases organ size and expands undifferentiated progenitor cells. *Curr Biol* 2007;17: 2054-2060.

2. Weiler SME, Pinna F, Wolf T, Lutz T, Geldiyev A, Sticht C, Knaub M, Thomann S, Bissinger M, Wan S, Rossler S, Becker D, et al. Induction of Chromosome Instability by Activation of Yes-Associated Protein and Forkhead Box M1 in Liver Cancer. *Gastroenterology* 2017;152: 2037-2051 e2022.

3. Guzman C, Bagga M, Kaur A, Westermarck J, Abankwa D. ColonyArea: an ImageJ plugin to automatically quantify colony formation in clonogenic assays. *PLoS One* 2014;9: e92444.

4. Pellegrino R, Castoldi M, Ticconi F, Skawran B, Budczies J, Rose F, Schwab C, Breuhahn K, Neumann UP, Gaisa NT, Loosen SH, Luedde T, et al. LINC00152 Drives a Competing Endogenous RNA Network in Human Hepatocellular Carcinoma. *Cells* 2022;11.

5. Fornes O, Castro-Mondragon JA, Khan A, van der Lee R, Zhang X, Richmond PA, Modi BP, Correard S, Gheorghe M, Baranasic D, Santana-Garcia W, Tan G, et al. JASPAR 2020: update of the open-access database of transcription factor binding profiles. *Nucleic Acids Res* 2020;48: D87-D92.

6. Rueden CT, Schindelin J, Hiner MC, DeZonia BE, Walter AE, Arena ET, Eliceiri KW. ImageJ2: ImageJ for the next generation of scientific image data. *BMC bioinformatics* 2017;18: 529.

7. Berg S, Kutra D, Kroeger T, Straehle CN, Kausler BX, Haubold C, Schiegg M, Ales J, Beier T, Rudy M, Eren K, Cervantes JI, et al. ilastik: interactive machine learning for (bio)image analysis. *Nat Methods* 2019;16: 1226-1232.

8. Schindelin J, Arganda-Carreras I, Frise E, Kaynig V, Longair M, Pietzsch T, Preibisch S, Rueden C, Saalfeld S, Schmid B, Tinevez JY, White DJ, et al. Fiji: an open-source platform for biological-image analysis. *Nat Methods* 2012;9: 676-682.
